# Simulating longitudinal data from marginal structural models using the additive hazard model
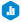


**DOI:** 10.1002/bimj.202000040

**Published:** 2021-05-13

**Authors:** Ruth H. Keogh, Shaun R. Seaman, Jon Michael Gran, Stijn Vansteelandt

**Affiliations:** 1 Department of Medical Statistics, London School of Hygiene & Tropical Medicine, London, UK; 2 MRC Biostatistics Unit, University of Cambridge, Institute of Public Health, Forvie Site, Robinson Way, Cambridge, UK; 3 Oslo Centre for Biostatistics and Epidemiology, Department of Biostatistics, Institute of Basic Medical Sciences, University of Oslo, Blindern, Oslo, Norway; 4 Department of Applied Mathematics, Computer Science and Statistics, Ghent University, Ghent, Belgium

**Keywords:** additive hazard model, causal inference, congenial models, longitudinal data, marginal structural model, simulation study, survival analysis, time-dependent confounding

## Abstract

Observational longitudinal data on treatments and covariates are increasingly used to investigate treatment effects, but are often subject to time-dependent confounding. Marginal structural models (MSMs), estimated using inverse probability of treatment weighting or the g-formula, are popular for handling this problem. With increasing development of advanced causal inference methods, it is important to be able to assess their performance in different scenarios to guide their application. Simulation studies are a key tool for this, but their use to evaluate causal inference methods has been limited. This paper focuses on the use of simulations for evaluations involving MSMs in studies with a time-to-event outcome. In a simulation, it is important to be able to generate the data in such a way that the correct forms of any models to be fitted to those data are known. However, this is not straightforward in the longitudinal setting because it is natural for data to be generated in a sequential conditional manner, whereas MSMs involve fitting marginal rather than conditional hazard models. We provide general results that enable the form of the correctly specified MSM to be derived based on a conditional data generating procedure, and show how the results can be applied when the conditional hazard model is an Aalen additive hazard or Cox model. Using conditional additive hazard models is advantageous because they imply additive MSMs that can be fitted using standard software. We describe and illustrate a simulation algorithm. Our results will help researchers to effectively evaluate causal inference methods via simulation.

## 1 Introduction

Observational longitudinal data are increasingly used to investigate the effects of treatments and exposures on health outcomes. To estimate treatment effects from observational data we must account for confounding of the treatment-outcome association, sometimes referred to as ‘confounding by indication’, and recent years have seen huge developments in statistical and epidemiological methods for this task. In this paper, we focus on the setting of estimating the joint effects of treatment across time-points on a time-to-event outcome using longitudinal data on treatment use and covariates, where time-dependent confounding is a specific challenge. When there is time-dependent confounding, standard analysis methods, such as Cox regression with adjustment for baseline or time-updated covariates, do not in general enable estimation of the causal effects of interest (Daniel et al., 2013).

Several methods have been described for estimating the causal effects of longitudinal treatment regimes on time-toevent outcomes. Marginal structural models (MSM) estimated using inverse probability of treatment weighting (IPTW) for time-to-event outcomes were introduced by Hernán et al. (2000), who described use of marginal structural Cox models (Cox MSM). Other methods include estimation of MSMs using the g-formula (also called g-computation) (Robins, 1986; Daniel et al., 2011; Keil et al., 2014), structural nested accelerated failure time models (Robins, 1992; Hernán et al., 2005), structural nested failure time models (Robins et al., 1992; Vansteelandt and Joffe, 2014), structural nested cumulative failure time models (Picciotto et al., 2012), and structural nested cumulative survival time models (Seaman et al., 2019). A recent review (Clare et al., 2018) found that among these, the Cox MSM approach is by far the most commonly used method in practice.

With the increasing development of more advanced causal inference methods, it is important to be able to evaluate method performance in different scenarios and make comparisons between methods to guide their use in practice. Simulation studies are a key tool for such investigations and can be used to assess properties such as bias, efficiency and coverage of confidence intervals. The results help analysts to choose which methods are most appropriate for answering research questions using their data. The importance of well-conducted simulation studies was highlighted by Morris et al. (2019), who provide detailed guidance for their planning and reporting. In this paper, we focus on the use of simulation studies for evaluations involving MSMs in the setting of a time-to-event outcome using longitudinal data on treatment use and covariates. When conducting a simulation study, it is desirable to be able to generate the data in such a way that the correct form of any analysis model to be fitted to those data is known, so that we know that the analysis model is correctly specified. For example, suppose that we wished to use a simulation study to assess the performance of the IPTW estimation approach for MSMs when the models for the weights are mis-specified in some way. It would be important to know that the MSM itself is correctly specified, so that any bias in the estimates can be attributed to mis-specification of the models used for the weights. As a second example, suppose that we wished to use a simulation study to compare the relative efficiency of the estimates of survival probabilities obtained using IPTW and using the g-formula. To make a fair comparison, the models involved in each approach should be correctly specified.

Generating longitudinal and time-to-event data in such a way that the forms of models used in methods applied to the data are known is not straightforward. A reason for this is that it is natural for the data to be generated in a sequential conditional manner, generating each individual’s covariates, treatment status, and survival status at each measurement time in turn conditional on the past, starting at time zero. This makes use of conditional models, including conditional hazard models for the time-to-event component. Analysis methods based on MSMs, on the other hand, make use of marginal (population average) rather than conditional hazard models. In this paper, we provide general results that enable the form of the correctly specified MSM for estimating causal treatment effects to be derived from an underlying conditional hazard model used in the data simulation procedure, and show how the results can be applied when the conditional hazard model is an additive hazard model (Aalen, 1989; Aalen et al., 2008) or a Cox model (Cox, 1972). We show that there is an advantage to using conditional additive hazard models for the data simulation, because this results in an additive form for the MSM, which can be fitted using standard software. The same does not hold for the Cox model. Martinussen and Vansteelandt (2013) provided results on the relation between conditional and marginal Cox models and conditional and marginal additive hazard models in the point-treatment setting. This paper extends their results to the longitudinal setting with time-dependent confounding. Young and Tchetgen Tchetgen (2014) investigated compatibility between conditional and marginal Cox models in the longitudinal and discrete-event-time setting under certain assumptions, but did not consider additive hazard models. We make use of earlier work on the g-formula (Robins, 1986; Keil et al., 2014; Daniel et al., 2013) to provide general results on the derivation of marginal hazard models based on an underlying conditional hazard model for a very general situation with time-dependent confounding.

The primary aim of this paper is to show how to simulate longitudinal data on treatments and covariates together with a time-to-event outcome in such a way that the form of the MSM that specifies the marginal hazard of the outcome is known, and hence that we know or are able to derive the true values of its parameters and of causal estimands of interest such as risk differences or risk ratios. The general results that we provide concerning the relation between conditional and marginal hazard models are key to informing the simulation algorithm. Our results will help researchers to effectively evaluate causal inference methods via simulation; a task of high importance but which is currently very rarely performed. Havercroft and Didelez (2012), Young et al. (2010),and Young and Tchetgen Tchetgen (2014) outlined algorithms for simulating longitudinal and time-to-event data to correspond with a specified Cox MSM, but their methods require restrictive assumptions about longitudinal relationships between variables or about distributions of variables, therefore limiting the simulation scenarios that can be generated—we discuss this earlier work in Section 7. We instead place an emphasis on use of additive hazard models, and the scenarios to which our results can be used are not limited, as in the earlier work.

The paper is organised as follows. In Section 2, we outline the longitudinal data set up and the notation. In Section 3, we review briefly why standard methods of analysis based on regression adjustment do not estimate the causal effects of interest and describe the use of MSMs in causal inference. Our main results are presented in Section 4, where we derive the relationship between a conditional hazard model and an MSM for the hazard and show the advantages of simulating data using an additive hazard model. In Section 5, we provide an example simulation algorithm and the algorithm is illustrated in Section 6. R code corresponding to the algorithm and the illustration is provided at https://github.com/ruthkeogh/causal_sim. We conclude with a discussion in Section 7.

## 2 Longitudinal Data and Time-Dependent Confounding

**Figure 1:**
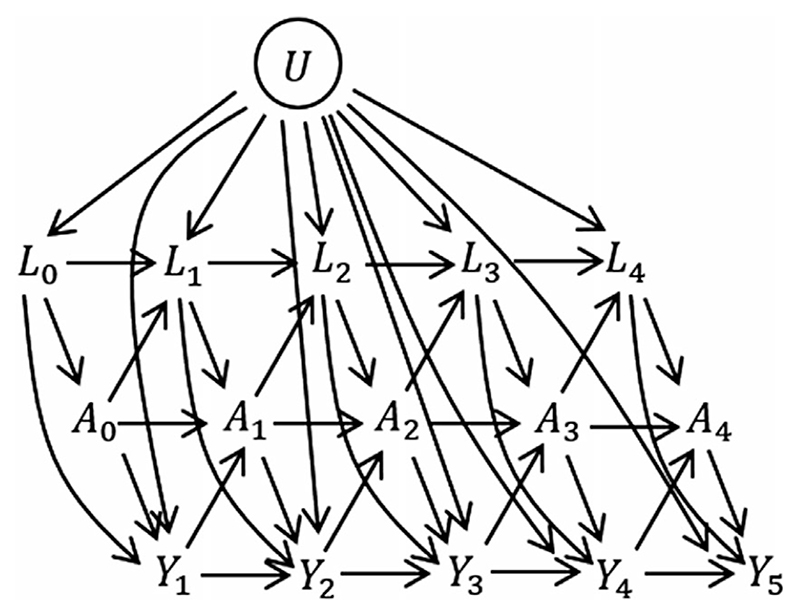
Causal directed acyclic graph (DAG) illustrating relationships between treatment *A*, time-dependent covariates *L*, an unmeasured frailty term *U* and time-to-event, illustrated for a discrete-time setting where *Y_k_* = *I*(*T* > *K*)

We consider a study in which *n* individuals are observed at regular visits up until the earlier of the time of the event of interest and the censoring time. The visit times, assumed to be the same for everybody, are *k* = 0,1, …,*K*. At each visit we observe binary treatment status *A_k_* and a set of time-dependent covariates *L_k_*. A bar over a time-dependent variable indicates the history, that is ${\bar{A}}_{k}=\left\{A_{0},A_{1},\ldots ,A_{k}\right\}$ and ${\bar{L}}_{k}=\left\{L_{0},L_{1},\ldots ,L_{k}\right\}$. We let ${\underline{A}}_{k}=\left\{A_{k},A_{k+1},\ldots ,A_{K}\right\}$ denote treatment from visit *k* up to *K*. The event time is denoted *T*. For simplicity we assume that all censoring is administrative at time *K* + 1, but the analysis methods that we focus on in this paper also accommodate loss to-follow-up and we discuss this in Section 7. Temporal causal relationships between variables are illustrated using a directed acyclic graph (DAG) in Figure 1. In the DAG the relationships are illustrated for a discrete-time setting where *Y_k_* = *I*(*T* > *k*). One can imagine extending the DAG by adding a series of small time intervals between each visit, at which *I*(*T* > *t*) is observed. As the time intervals become very small we approach the continuous time setting. The DAG also includes a variable *U*, which has direct effects on *L_k_* and *Y_k_* but not on *A_k_*. *U* is an unmeasured individual frailty and we include it because it is realistic that such individual frailty effects exist in practice. Because *U* is not a confounder of the assocation between *A_k_* and the outcome *T*, the fact that it is unmeasured does not affect our ability to estimate causal effects of treatments.

It is possible to use the longitudinal data to estimate the impact of treatment at visit *k*, *A_k_* on the concurrent hazard, for example using a Cox regression with time-updated treatment variable and with adjustment for confounding by the past treatment and covariate history, $\left({\bar{A}}_{k-1},{\bar{L}}_{k}\right)$. This is discussed in Section 3.1. However, questions about causal joint effects of treatments over time are more difficult to answer, due to the presence of time-dependent confounding. An example of a question about causal joint treatment effects is whether there is a difference in the probability of survival up to *τ* years had an individual been assigned by an intervention to have *A* = 1 at all time points versus had they been assigned to have *A* = 0 at all time points. Time-dependent confounding occurs when there are time-dependent covariates that predict subsequent treatment use, are affected by earlier treatment, and affect the outcome through pathways that are not just through subsequent treatment. The *L_k_* are time-dependent confounders in the DAG in Figure 1. The DAG could be extended in various ways, in particular so that there are long term effects of *L* on *A* and vice versa. For example, we could add arrows from *L_k_* to *A*_*k*+1_ and from *A_k_* to *L*_*k*+2_. Long term effects of *A* and *L* on survival could also be added, for example by adding arrows from *L_k_* and *A_k_* to *Y*_*k*+2_.

## 3 Estimating Treatment Effects Using Longitudinal Data

### 3.1 Traditional survival analysis

We beginbybrieflyreviewingtraditionalmethodsofanalysisforinvestigatingtheassociationbetweenatime-dependent treatment variable andatime-to-eventoutcome. Byfarthe most popular approach is Cox regression (Cox, 1972). Consider a Cox regression model in which the hazard at time *t*, incorporating time-dependent covariates, is

(1) $$\lambda \left(t|{\bar{A}}_{\left\lfloor t\right\rfloor },{\bar{L}}_{\left\lfloor t\right\rfloor }\right)=\lambda_{0}(t)\exp\left(\beta_{A0}A_{\left\lfloor t\right\rfloor }+I(t>1)\overset{\left\lfloor t\right\rfloor }{\underset{j=1}{\sum }}\beta_{Aj}A_{\lfloor t\rfloor -j}+\overset{\left\lfloor t\right\rfloor }{\underset{j=0}{\sum }}\beta_{Lj}L_{\lfloor t\rfloor -j}\right)$$

where $A_{\left\lfloor t\right\rfloor }$ and $L_{\left\lfloor t\right\rfloor }$ denote the values at the most recent visit prior to time *t* (in a slight abuse of standard notation, ⌊t⌋ is the largest integer less than *t*), *λ*_0_(*t*) is the baseline hazard, and the *β* parameters are log hazard ratios. The hazard ratio exp(*β*_*A*0_) is the instantaneous multiplicative effect of the current treatment $A_{\left\lfloor t\right\rfloor }$ on the hazard among individuals at risk at time *t*, assumed to be the same for all *t*, adjusted for past variables (including past treatment), which are confounders of the association between $A_{\left\lfloor t\right\rfloor }$ and the current hazard. The other model parameters do not have a straightforward interpretation. For example, the coefficient for $A_{\lfloor t\rfloor -1},\beta_{A1}$, is conditional on covariates that include $A_{\left\lfloor t\right\rfloor }$ and $L_{\left\lfloor t\right\rfloor }$, which are on the mediating pathway from $A_{\lfloor t\rfloor -1}$ to survival, and so its interpretation is complicated. Hence, the estimation of joint effects of treatments over time is not accommodated using the traditional Cox modelling approach with time-dependent covariates. Furthermore, a growing body of work has explained that hazard ratios do not have a straightforward causal interpretation (Hernán, 2010; Aalen et al., 2015; Martinussen et al., 2019) and so there are subtleties in the interpretation of *β*_*A*0_ even when all confounders have been included.

Aalen’s additive hazard model (Aalen, 1989; Aalen et al., 2008) has been much less used in practice, but its attractive properties are increasingly being recognised (Martinussen and Vansteelandt, 2013). Consider an additive hazard model in which the hazard at time *t*, incorporating time-dependent covariates, is

(2) $$\lambda \left(t|{\bar{A}}_{\left\lfloor t\right]},{\bar{L}}_{\left\lfloor t\right\rfloor }\right)=\alpha_{0}(t)+\alpha_{A0}(t)A_{\left\lfloor t\right]}+I(t>1)\overset{\left\lfloor t\right\rfloor }{\underset{j=1}{\sum }}\alpha_{Aj}(t)A_{\lfloor t\rfloor -j}+\overset{\left\lfloor t\right\rfloor }{\underset{j=0}{\sum }}\alpha_{Lj}(t)L_{\lfloor t\rfloor -j}$$

where the parameters *α*_0_(*t*), *α_Aj_*(*t*), *α_Lj_*(*t*) (*j* = 0, …, 4) are arbitrary functions of time, meaning that the model is fully non-parametric. The results from the additive hazard model are typically presented as cumulative coefficients, for example ${\int^{\text{​}}}_{0}^{t}\alpha_{A0}(s)ds$. The discussion above about the interpretation of *β*_*A*0_ is equally relevant to *α*_*A*0_(*t*), and again the presence of time-dependent confounding means that joint effects of treatments over time cannot be estimated directly from the traditional additive hazard model. An advantage of the additive hazard model relative to the Coxmodel is that the parameters of the additive hazard model are collapsible,meaning that the parameter associated with a given covariate in a given model has the same interpretation as that in a model which is additionally adjusted for variables that are not associated with that covariate (Martinussen and Vansteelandt, 2013). By contrast, hazard ratios are non-collapsible,meaning that the Cox model does not have this property. Collapsibility has implications for the relation between conditional models and marginal models. In Section 4, we use this property to show that a conditional additive hazard model, of a form such as that in (2), has a useful role in the simulation of longitudinal data in such a way that the form of the correctly specified MSM for the hazard is known.

### 3.2 Marginal structural hazard models

MSMs are models for counterfactual outcomes. We let $T^{{\underline{a}}_{0}}$ denote the counterfactual event time for a given individual had they followed treatment regime ${\underline{a}}_{0}$ from visit 0 onwards. The marginal hazard at time *t* under the possibly counter-to-fact treatment regime ${\underline{a}}_{0}$ is the hazard in the population if everyone were to follow that treatment regime, and is denoted $\lambda_{T^{{\underline{a}}_{0}}}(t)$.

In the context of time-to-event outcomes, the MSM is usually assumed to take the Cox proportional hazards form

(3) $$\lambda_{T^{{\underline{a}}_{0}}}(t)=\lambda_{0}(t)\exp\left\{g\left({\bar{a}}_{\left\lfloor t\right\rfloor };{\tilde{\beta }}_{A}\right)\right\}$$

where *λ*_0_(*t*) is the baseline counterfactual hazard, ${\bar{a}}_{\left\lfloor t\right\rfloor }$ denotes treatment pattern up to the most recent visit prior to time *t*, $g\left({\bar{a}}_{\left\lfloor t\right\rfloor };{\tilde{\beta }}_{A}\right)$ is a function (to be specified) of treatment pattern ${\bar{a}}_{\left\lfloor t\right\rfloor }$, and ${\tilde{\beta }}_{A}$ is a vector of log hazard ratios. The hazard model could take any form, however, and we also consider MSMs based on Aalen’s additive hazard model:

(4) $$\lambda_{T^{{\underline{a}}_{0}}}(t)={\tilde{\alpha }}_{0}(t)+g\left({\bar{a}}_{\left\lfloor t\right]};{\tilde{\alpha }}_{A}(t)\right)$$

The MSM must specify how the hazard at time *t* depends on the history of treatment up to time *t*, ${\bar{a}}_{\left\lfloor t\right\rfloor }$, through the function g(·). In a simple form for the MSM, the hazard at time *t* is specified to depend only on the current level of treatment, so that $g\left({\bar{a}}_{\left\lfloor t\right\rfloor };{\tilde{\beta }}_{A}\right)={\tilde{\beta }}_{A}a_{\left\lfloor t\right\rfloor }$ in the Cox MSM and $g\left({\bar{a}}_{\left\lfloor t\right\rfloor };{\tilde{\alpha }}_{A}(t)\right)={\tilde{\alpha }}_{A}(t)a_{\left\lfloor t\right\rfloor }$ in the Aalen MSM. Other examples are for the hazard at *t* to depend on duration of treatment, using $g\left({\bar{a}}_{\left\lfloor t\right\rfloor };{\tilde{\beta }}_{A}\right)={\tilde{\beta }}_{A}\sum_{j=0}^{\left\lfloor t\right\rfloor }a_{\lfloor t\rfloor -j}$ in the Cox MSM and $g\left({\bar{a}}_{\left\lfloor t\right\rfloor };{\tilde{\alpha }}_{A}(t)\right)={\tilde{\alpha }}_{A}(t)\sum_{j=0}^{\left\lfloor t\right\rfloor }a_{\lfloor t\rfloor -j}$ in the Aalen MSM, or on the history of treatment through main effect terms for treatment at each visit, using $g\left({\bar{a}}_{\left\lfloor t\right\rfloor };{\tilde{\beta }}_{A}\right)=\sum_{j=0}^{\left\lfloor t\right\rfloor }{\tilde{\beta }}_{Aj}a_{\lfloor t\rfloor -j}$ in the Cox MSM and $\text{g}\left({\bar{a}}_{\left\lfloor t\right\rfloor };{\tilde{\alpha }}_{A}(t)\right)=\sum_{j=0}^{\left\lfloor t\right\rfloor }{\tilde{\alpha }}_{Aj}(t)a_{\lfloor t\rfloor -j}$ in the Aalen MSM.

When there is confounding an MSM cannot be estimated by fitting the model to the observed data using standard regression. The most commonly used estimation approach uses IPTW, in which individuals are reweighted using time-dependent weights (Daniel et al., 2013; Cole and Hernán, 2008). Further details on the weights are given in the Supporting Information (Section A1). MSMs can also be estimated using the g-formula (Robins, 1986; Daniel et al., 2011), and the methods described in Section 4 make use of this. The use of MSMs estimated using these methods to estimate causal effects of joint treatments over time involves the four key assumptions of no interference, positivity, consistency, and conditional exchangeability (no unmeasured confounding) (Robins et al., 2000; VanderWeele, 2009;Daniel et al., 2013). The no interference assumption is that the counterfactual event time for a given individual, $T^{{\underline{a}}_{0}}$, does not depend on the treatment received by any other individuals. The positivity assumption is that each individual has a strictly non-zero probability of receiving each given pattern of treatments over time. Consistency means that an individual’s observed outcome is equal to the counterfactual outcome when the assigned treatment pattern is set to that which was actually received, i.e. $T_{i}=T_{i}^{{\underline{A}}_{0,l}}$. The conditional exchangeability assumption can be expressed formally as $T^{{\bar{A}}_{k-1},a_{k}}\underline{∥}A_{k}|{\bar{A}}_{k-1},{\bar{L}}_{k},T\geq k$ for all feasible ${\underline{a}}_{k}$, where $T^{{\bar{A}}_{k-1},{\underline{a}}_{k}}$ denotes the counterfactual event time had an individual followed their observed treatment pattern up to time *k* − 1, ${\bar{A}}_{k-1}$, and had their treatments been set to ${\underline{a}}_{k}$ from time *k* onwards, given survival to time *k*. The conditional exchangeability assumption means that among individuals who remain at risk of the event at time *k*, the treatment *A_k_* received at time *k* may depend on past treatment and covariates ${\bar{A}}_{k-1}$ and ${\bar{L}}_{k}$, but that, conditional on these, it does not depend on the remaining lifetime that would apply if all future treatments were set to any particular values ${\underline{a}}_{k}$.

The Cox MSM gives rise to estimates of the log hazard ratios ${\tilde{\beta }}_{A}$, and the Aalen MSM to estimates of cumulative regression coefficients ${\int^{\text{​}}}_{0}^{t}{\tilde{\alpha }}_{A}(s)ds$. As noted in Section 3.1, hazard-based estimands, which include hazard ratios from a Cox model and cumulative regression coefficients from Aalen’s additive hazard model, have been shown not to have a direct causal interpretation. Therefore, it is desirable to translate the estimates from the MSM into an estimate for a causal estimand such as a risk difference or a risk ratio. For example, the marginal risk difference at time *t* had all individuals been treated up to time *t* versus had all individuals not been treated up to time *t* is $\mathrm{Pr}\left(T^{{\underline{a}}_{0}}{\;}^{=1}\geq t\right)-\mathrm{Pr}\left(T^{{\underline{a}}_{0}}{\;}^{=0}>t\right)$Based on the Cox MSM in (3), the counterfactual survival probability at time *t* is

(5) $$\mathrm{Pr}(T^{{\underline{a}}_{0}}\geq t)=\exp\left(-e^{g\left(a_{0};\beta_{A}\right)}{\int^{\text{​}}}_{0}^{1}\lambda_{0}(s)ds-e^{g\left({\bar{a}}_{1};\beta_{A}\right)}{\int^{\text{​}}}_{1}^{2}\lambda_{0}(s)ds\cdots -e^{g\left({\bar{a}}_{\left\lfloor t\right\rfloor };\beta_{A}\right)}{\int^{\text{​}}}_{\left\lfloor t\right\rfloor }^{t}\lambda_{0}(s)ds\right)$$

where the baseline cumulative hazard can be estimated using (an inverse probability weighted) Breslow’s estimator. The counterfactual survival probability based on the Aalen MSM in (4) is

(6) $$\mathrm{Pr}\left(T^{{\underline{a}}_{0}}\geq t\right)=\exp\left(-{\int^{\text{​}}}_{0}^{t}{\tilde{\alpha }}_{0}(s)ds-{\int^{\text{​}}}_{0}^{1}g\left(a_{0};{\tilde{\alpha }}_{A}(s)\right)ds-{\int^{\text{​}}}_{1}^{2}g\left({\bar{a}}_{1};{\tilde{\alpha }}_{A}(s)\right)ds\cdots -{\int^{\text{​}}}_{\left\lfloor t\right\rfloor }^{t}g\left({\bar{a}}_{\left\lfloor t\right\rfloor };{\tilde{\alpha }}_{A}(s)\right)ds\right)$$

## 4 Simulation From MSMs

As noted in Section 1, when conducting a simulation study to evaluate and compare the properties of analysis methods, it is important to be able to generate the data in such a way that the forms of any models to be estimated using the simulated data are known based on the data generating mechanism. In our context, for evaluations involving MSMs it is therefore important to know the correct form of the MSM, and hence know or be able to derive the true values of its parameters and of causal estimands of interest such as risk differences or risk ratios. Itmay also be of interest in some contexts to evaluate the impact of using a mis-specfied MSM, in which case we need to understand how the model under consideration differs from the correctly specified MSM.

When simulating longitudinal and time-to-event data, such as for the situation depicted in the DAG in Figure 1, it is natural to generate the data sequentially in time. We provide a detailed algorithm in Section 5. Briefly, the procedure starts by generating *U*, then *L*_0_|*U*, then *A*_0_|*L*_0_, *U*, and then event times in period 0 < *t* < 1 using the hazard *λ*(*t*|*A*_0_, *L*_0_, *U*). The next step is to generate *L*_1_|*A*_0_, *L*_0_, *U*,*T* ≥ 1, followed by *A*_1_|*A*_0_, *L*_0_, *L*_1_, *U*,*T* ≥ 1, and then event times in period 1 ≤ *t* < 2 using the hazard *λ*(*t*|*A*_0_, *A*_1_, *L*_0_, *L*_1_, *U*). Analogous steps are then carried out for each of visits 2, 3 and so on up to *K*. This procedure uses the conditional hazards $\lambda \left(t|{\bar{A}}_{\lfloor },{\bar{L}}_{\lfloor },U\right)$. The MSM describes instead the marginal hazard, which is a function only of the assigned treatment up to time *t*, ${\bar{a}}_{\left\lfloor t\right\rfloor }$, and not of ${\bar{L}}_{\left\lfloor t\right\rfloor }$ or *U*. The question therefore arises as to what the form of the MSM is under the sequential data generating procedure outlined above, which uses a conditional hazard model and conditional models for the time-dependent covariates.

### 4.1 Link between conditional and marginal hazard models

In this section we derive general results for the link between the conditional models used to simulate the longitudinal and time-to-event data and the MSM $\lambda_{T^{{\underline{a}}_{0}}}(t)$. These general results are then used in the context of additive hazard models and Cox models. This extends some of the work of Martinussen and Vansteelandt (2013) to the longitudinal setting. Our overall approach is to first use the g-formula for time-to-event outcomes (Robins, 1986; Keil et al., 2014; Daniel et al., 2013) to express the survivor function for counterfactual event times, $\mathrm{Pr}\left(T^{{\underline{a}}_{0}}\geq t\right)$, in terms of conditional distributions of observed event times and variables *A*, *L*, *U*, and then use the fact that the hazard can be expressed as minus the derivative of the log of the survivor function: $\lambda_{T^{{\underline{a}}_{0}}}(t)=\frac{-\frac{d}{dt}\mathrm{Pr}\left(T^{{\underline{a}}_{0}}\geq t\right)}{\mathrm{Pr}\left(T^{{\underline{a}}_{0}}\geq t\right)}$. We first consider the effect of treatment at time 0, *a*_0_, on the hazard at times 0 < *t* < 1, and then the effect of treatment at times 0 and 1 on the hazard at times 1 ≤ *t* < 2, and so on.

The DAG in Figure 1 is just one example of a situation to which the general results given in this section apply. The results also apply for extended settings, noted in Section 2, in which there could be longer term effects of *L* on *A* and vice versa, and longer term effects of *A* and *L* on the hazard. The results do not rely on the existence of the unobserved variable *U*. We focus on a setting in which events are observed in continuous time.

By averaging over *L*_0_ and *U*, the marginal survival probability $\mathrm{Pr}\left(T^{{\underline{a}}_{0}}\geq t\right)$ for 0 < *t* < 1 can be expressed as

(7) $$\begin{array}{lll} \mathrm{Pr}\left(T^{{\underline{a}}_{0}}\geq t\right) & = & \int^{\text{​}}\mathrm{Pr}\left(T^{\underline{a}}0\geq t|L_{0},U\right)f\left(L_{0},U\right)dL_{0}dU\\ \; & = & \int^{\text{​}}\mathrm{Pr}\left(T\geq t|A_{0}=a_{0},L_{0},U\right)f\left(L_{0},U\right)dL_{0}dU \end{array}$$

where the second line follows from the conditional exchangeability assumption $T^{{\underline{a}}_{0}}\underline{∥}A_{0}|L_{0}$ and consistency. Using the relation between the hazard and the survivor function the hazard corresponding to the survival function in (7) can be written

(8) $$\begin{array}{lll} \lambda_{T^{{\underline{a}}_{0}}}(t) & = & \frac{-\int^{\text{​}}\frac{d}{dt}\mathrm{Pr}\left(T\geq t|A_{0}=a_{0},L_{0},U\right)f\left(L_{0},U\right)dL_{0}dU}{\int^{\text{​}}\mathrm{Pr}\left(T\geq t|A_{0}=a_{0},L_{0},U\right)f\left(L_{0},U\right)dL_{0}dU}\\ \; & = & \frac{\int^{\text{​}}\lambda \left(t|A_{0}=a_{0},L_{0},U\right)\mathrm{Pr}\left(T\geq t|A_{0}=a_{0},L_{0},U\right)f\left(L_{0},U\right)dL_{0}dU}{\int^{\text{​}}\mathrm{Pr}\left(T\geq t|A_{0}=a_{0},L_{0},U\right)f\left(L_{0},U\right)dL_{0}dU}\\ \; & = & \frac{E_{L_{0},U}\left\{\lambda \left(t|A_{0}=a_{0},L_{0},U\right)\mathrm{Pr}\left(T\geq t|A_{0}=a_{0},L_{0},U\right)\right\}}{E_{L_{0},U}\left\{\mathrm{Pr}\left(T\geq t|A_{0}=a_{0},L_{0},U\right)\right\}}\\ \; & = & \frac{E_{L_{0},U}\left\{\lambda \left(t|A_{0}=a_{0},L_{0},U\right)\exp\left(-{\int^{\text{​}}}_{0}^{t}\lambda \left(s|A_{0}=a_{0},L_{0},U\right)ds\right)\right\}}{E_{L_{0},U}\left\{\exp\left(-{\int^{\text{​}}}_{0}^{t}\lambda \left(s|A_{0}=a_{0},L_{0},U\right)ds\right)\right\}} \end{array}$$

where *E*_*L*_0_,U_(·) denotes the expectation over the joint distribution of *L*_0_ and *U*. For 0 < *t* < 1, the MSM $\lambda_{T^{{\underline{a}}_{0}}(t)}$ can therefore be expressed as a function of the conditional hazard *λ*(*t*|*A*_0_ = *a*_0_, *L*_0_, *U*) and conditional distributions of variables *A*_0_, *L*_0_, *U*.

Next, we derive an expression for the marginal survivor function $\mathrm{Pr}\left(T^{{\underline{a}}_{0}}\geq t\right)$ for 1 ≤ *t* < 2, followed by an expression for the corresponding hazard. To derive the survivor function, first consider averaging over the baseline variables *L*_0_ and *U*:

(9) $$\begin{array}{lll} \mathrm{Pr}\left(T^{{\underline{a}}_{0}}\geq t\right) & = & \int^{\text{​}}\mathrm{Pr}\left(T^{{\underline{a}}_{0}}\geq t|A_{0}=a_{0},L_{0},U,T^{{\underline{a}}_{0}}\geq 1\right)\mathrm{Pr}\left(T^{{\underline{a}}_{0}}\geq 1|A_{0}=a_{0},L_{0},U\right)f\left(L_{0},U\right)dL_{0}dU\\ \; & = & \int^{\text{​}}\mathrm{Pr}\left(T^{{\underline{a}}_{0}}\geq t|A_{0}=a_{0},L_{0},U,T\geq 1\right)\mathrm{Pr}\left(T\geq 1|A_{0}=a_{0},L_{0},U\right)f\left(L_{0},U\right)dL_{0}dU. \end{array}$$

The second line follows because the events that $T^{{\underline{a}}_{0}}\geq 1$ and *T* ≥ 1 are the same for individuals with *A*_0_ = *a*_0_. In the next step we first average over *L*_1_|*L*_0_, *U*, *T* ≥ 1 and then use the conditional exchangeability assumption $T^{{\underline{a}}_{0}}\underline{∥}A_{1}|{\bar{L}}_{1}$, *A*_0_ = *a*_0_, *T* ≥ 1 and consistency to give

(10) $$\begin{array}{lll} \mathrm{Pr}(T^{{\underline{a}}_{0}}\geq t) & = & \int^{\text{​}}\mathrm{Pr}\left(T^{{\underline{a}}_{0}}\geq t|A_{0}=a_{0},{\bar{L}}_{1},U,T\geq 1\right)\mathrm{Pr}\left(T\geq 1|A_{0}=a_{0},L_{0},U\right)\\ \; & \; & \times f\left(L_{1}|A_{0}=a_{0},L_{0},U,T\geq 1\right)f\left(L_{0},U\right)d{\bar{L}}_{1}dU\\ \; & = & \int^{\text{​}}\mathrm{Pr}\left(T\geq t|A_{0}=a_{0},A_{1}=a_{1},{\bar{L}}_{1},U,T\geq 1\right)\mathrm{Pr}\left(T\geq 1|A_{0}=a_{0},L_{0},U\right)\\ \; & \; & \times f\left(L_{1}|A_{0}=a_{0},L_{0},U,T\geq 1\right)f\left(L_{0},U\right)d{\bar{L}}_{1}dU\\ \; & = & E_{L_{0},U}\left[E_{L_{1}|A_{0}=a_{0},L_{0},U,T\geq 1}\left\{\mathrm{Pr}\left(T\geq t|{\bar{A}}_{1}={\bar{a}}_{1},{\bar{L}}_{1},U,T\geq 1\right)\mathrm{Pr}\left(T\geq 1|A_{0}=a_{0},L_{0},U\right)\right\}\right] \end{array}$$

Finally, using the relation between the hazard and survivor function it can be shown that for 1 ≤ *t* < 2

(11) $$\lambda_{T^{{\underline{a}}_{0}}}(t)=\frac{E_{L_{0},U}[E_{L_{1}|A_{0}=a_{0},L_{0},U,T\geq 1}\left\{\lambda \left(t|{\bar{A}}_{1}={\bar{a}}_{1},{\bar{L}}_{1},U\right)\exp\left(-{\int^{\text{​}}}_{0}^{1}\lambda \left(s|A_{0}=a_{0},L_{0},U\right)ds-{\int^{\text{​}}}_{1}^{t}\lambda \left(s|{\bar{A}}_{1}={\bar{a}}_{1},{\bar{L}}_{1},U\right)ds\right)\right\}]}{E_{L_{0},U}[E_{L_{1}|A_{0}=a_{0},L_{0},U,T\geq 1}\left\{\exp\left(-{\int^{\text{​}}}_{0}^{1}\lambda \left(s|A_{0}=a_{0},L_{0},U\right)ds-{\int^{\text{​}}}_{1}^{t}\lambda \left(s|{\bar{A}}_{1}={\bar{a}}_{1},{\bar{L}}_{1},U\right)ds\right)\right\}]}$$

It follows that for 1 ≤ *t* < 2 the MSM $\lambda_{T^{{\underline{a}}_{0}}}(t)$ can be expressed as a function of the conditional hazard $\lambda \left(t|{\bar{A}}_{1},{\bar{L}}_{1},U\right)$ and conditional distributions of variables ${\bar{A}}_{1},{\bar{L}}_{1},U$.

A general expression for the MSM at times *k* ≤ *t* < *k* + 1 is

(12) $$\lambda_{T^{{\underline{a}}_{0}}}(t)=\frac{E_{L_{0},U}[E_{L_{1}|A_{0}=a_{0},L_{0},U,T\geq 1}\left\{\cdots E_{L_{k}|{\bar{A}}_{k-1}={\bar{a}}_{k-1},{\bar{L}}_{k-1},U,T\geq k}\left(\lambda \left(t|{\bar{A}}_{k}={\bar{a}}_{k},{\bar{L}}_{k},U,\right)Q_{k}\right)\right\}]}{E_{L_{0},U}[E_{L_{1}|A_{0}=a_{0},L_{0},U,T\geq 1}\left\{\cdots E_{L_{k}\left|{\bar{A}}_{k-1}\right|}^{|}{\bar{a}}_{k-1},L_{k-1},U,T\geq k\left(Q_{k}\right)\right\}]}$$

where $Q_{k}=\overset{j=0}{\underset{k-1}{\prod^{\text{​}}}}\exp(-{\int^{\text{​}}}_{j}^{j+1}\lambda \left(s|{\bar{A}}_{j}=a_{j},{\bar{L}}_{j},U\right)ds)\exp\left(-{\int^{\text{​}}}_{k}^{t}\lambda \left(s|{\bar{A}}_{k}=a_{k},{\bar{L}}_{k},U\right)ds\right)$.

The above results show how the MSM $\lambda_{T^{{\underline{a}}_{0}}}(t)$ can be expressed in terms of the conditional hazard model for the observed data, $\lambda \left(t|{\bar{A}}_{\left\lfloor t\right\rfloor },{\bar{L}}_{\left\lfloor t\right\rfloor },U\right)$, and conditional distributions for the observed time-dependent covariates. The results were derived by making use of the g-formula. We next apply these results to the situations in which the conditional hazard model $\lambda \left(t|{\bar{A}}_{\left\lfloor t\right\rfloor },{\bar{L}}_{\left\lfloor t\right\rfloor },U\right)$ follows an Aalen additive hazard model or a Cox model.

### 4.2 Results using conditional additive hazard models

Suppose that the conditional hazard model is of the additive form

(13) $$\lambda \left(t|{\bar{A}}_{\left\lfloor t\right\rfloor },{\bar{L}}_{\left\lfloor t\right\rfloor },U\right)=\alpha_{0}(t)+\alpha_{A}^{⊤}(t)v\left({\bar{A}}_{\left\lfloor t\right\rfloor }\right)+\alpha_{L}^{⊤}(t)w\left({\bar{L}}_{\left\lfloor t\right\rfloor }\right)+\alpha_{U}(t)U$$

where *α_A_*(*t*) and *α_L_*(*t*) are vectors of parameters and the hazard at time *t* depends on a known vector function of ${\bar{A}}_{\left\lfloor t\right\rfloor }$, $v\left({\bar{A}}_{\left\lfloor t\right\rfloor }\right)$, and a known vector function of ${\bar{L}}_{\left\lfloor t\right\rfloor },w\left({\bar{L}}_{\left\lfloor t\right\rfloor }\right)$.

It can be shown that $\lambda_{T^{{\underline{a}}_{0}}}(t)$ also takes the form of an additive hazard model in this case. We provide results for 0 < *t* < 1 and 1 ≤ *t* < 2 to illustrate the point. For 0 < *t* < 1, using the general expression in (8), we have

(14) $$\lambda_{T^{{\underline{a}}_{0}}}(t)=\alpha_{0}(t)+\alpha_{A}^{⊤}(t)v\left(a_{0}\right)+\frac{E_{L_{0},U}[E_{L_{1}|A_{0}=a_{0},L_{0},U,T\geq 1}\left\{\left(\alpha_{L}^{⊤}(t)w\left({\bar{L}}_{1}\right)+\alpha_{U}(t)U\right)ℛ\left({\bar{L}}_{1},U\right)\right\}]}{E_{L_{0},U}\left\{\exp\left(-{\int^{\text{​}}}_{0}^{t}\left(\alpha_{L}^{⊤}(s)w\left(L_{0}\right)+\alpha_{U}(s)U\right)ds\right)\right\}}$$

This expression for $\lambda_{T^{{\underline{a}}_{0}}}(t)(0<t<1)$ is of the additive form, $\lambda_{T^{{\underline{a}}_{0}}}(t)={\tilde{\alpha }}_{0}(t)+\alpha_{A}^{⊤}(t)v\left(a_{0}\right)$. The coefficient for *υ*(*α*_0_), *α_A_*(*t*), is the same as in the conditional hazard model, whereas the intercept ${\tilde{\alpha }}_{0}(t)$ is now the sum of *α*_0_(*t*) and the third term in the expression in (14). Note that since the treatment is binary *υ*(*a*_0_) = *a*_0_ (or, trivially, *υ*(*a*_0_) = 1 if the conditional hazard (13) at times 0 < *t* < 1 does not depend on *A*_0_). The result in (14) is similar to that derived by Martinussen and Vansteelandt (2013), who considered the form of the marginal hazard in the setting of a point treatment, except they did not incorporate a *U* variable.

For 1 ≤ *t* < 2 it can be shown using (11) that the MSM is of the form

(15) $$\lambda_{T^{{\underline{a}}_{0}}}(t)=\alpha_{0}(t)+\alpha_{A}^{⊤}(t)v\left({\bar{a}}_{1}\right)+\frac{E_{L_{0},U}[E_{L_{1}|A_{0}=a_{0},L_{0},U,T\geq 1}\left\{\left(\alpha_{L}^{⊤}(t)w\left({\bar{L}}_{1}\right)+\alpha_{U}(t)U\right)ℛ\left({\bar{L}}_{1},U\right)\right\}]}{E_{L_{0},U}\left[E_{L_{1}|A_{0}=a_{0},L_{0},U,T\geq 1}\left\{ℛ\left({\bar{L}}_{1},U\right)\right\}\right]}$$

where $ℛ\left({\bar{L}}_{1},U\right)=\exp\left(-{\int^{\text{​}}}_{0}^{1}\left(\alpha_{L}^{⊤}(s)w\left(L_{0}\right)+\alpha_{U}(s)U\right)ds-{\int^{\text{​}}}_{1}^{t}\left(\alpha_{L}^{⊤}(s)w\left({\bar{L}}_{1}\right)+\alpha_{U}(s)U\right)ds\right)$. The third term of (15) is a function of *a*_0_. It follows from this expression that for a binary treatment the MSM $\lambda_{T^{{\underline{a}}_{0}}}(t)$ is of the additive hazard form $\lambda_{T^{{\underline{a}}_{0}}}(t)={\tilde{\alpha }}_{0}(t)+\alpha_{A}^{⊤}(t)v\left({\bar{a}}_{1}\right)+{\tilde{\alpha }}_{A}^{*}(t)a_{0}$. In the setting where $\alpha_{A}^{⊤}(t)v\left({\bar{a}}_{1}\right)=\alpha_{A0}(t)a_{1}+\alpha_{A1}(t)a_{0}$, the coefficient for *a*_1_ in the MSM $\lambda_{T^{{\underline{a}}_{0}}}(t)(1\leq t<2)$ is the same as that in the conditional hazard model, *α*_*A*0_(*t*), whereas the intercept and the coefficient for *a*_0_ are different from those in the conditional hazard model.

The result that the MSM $\lambda_{T^{{\underline{a}}_{0}}}(t)$ is of an additive form when the conditional hazard model is an additive model does not rely on distributional assumptions for *L* and *U*. Finding expressions for the third terms in (14) and (15) (ratios of nested expectations) is in general intractable (see Supporting Information Section A2 for an example where closed form expressions are available). In Section 6.2, we describe a general simulation-based approach to deriving the true values of the parameters of the MSM, which is straightforward to implement.

In the conditional additive hazard model in (13) the treatment history is included in the general form$v\left({\bar{A}}_{\left\lfloor t\right]}\right)$. In practice, as discussed in Section 3.2, this form has to be specified. Suppose that the conditional hazard model was of a form such that the hazard at time *t* depends only on the current treatment status $A_{\left\lfloor t\right\rfloor }$, that is $\lambda \left(t|{\bar{A}}_{\left|t\right|},{\bar{L}}_{\left|t\right|},U\right)=\alpha_{0}(t)+\alpha_{A}(t)A_{\left|t\right|}+\alpha_{L}^{⊤}(t)w\left({\bar{L}}_{\left\lfloor t\right\rfloor }\right)+\alpha_{U}(t)U$. The result in (15) shows that even if the conditional hazard at time *t* (1 ≤ *t* < 2) depends on treatment only through the current level, *a*_1_, the MSM depends on both *a*_0_ and *a*_1_ for 1 ≤ *t* < 2. The intuition behind this result is that *A*_0_ affects *L*_1_ and hence after the averaging over *L*_1_, the marginal hazard at time *t* (1 ≤ *t* < 2) depends on *a*_0_. In general, even if the conditional hazard at time *t* depends on treatment only through the current level, $a_{\left\lfloor t\right\rfloor }$, the MSM depends on the whole history of treatment ${\bar{a}}_{\left\lfloor t\right\rfloor }$. In the Supporting Information (Section A3) we extend the results to the setting where the conditional hazard model (13) additionally includes interactions between ${\bar{A}}_{\left\lfloor t\right\rfloor }$ and ${\bar{L}}_{\left\lfloor t\right\rfloor }$.

### 4.3 Results using conditional Cox models

Suppose instead that the conditional hazard model is of the Cox proportional hazards form

(16) $$\lambda \left(t|{\bar{A}}_{\left\lfloor t\right]},{\bar{L}}_{\left\lfloor t\right\rfloor },U\right)=\lambda_{0}(t)\exp\left(\beta_{A}^{⊤}v\left({\bar{A}}_{\left\lfloor t\right]}\right)+\beta_{L}^{⊤}w\left({\bar{L}}_{\left\lfloor t\right\rfloor }\right)+\beta_{U}U\right)$$

For 0 < *t* < 1, using the general expression in (8), the MSM takes the form

(17) $$\lambda_{T^{{\underline{a}}_{0}}}(t)=\lambda_{0}(t)\exp\left(\beta_{A}^{⊤}v\left(a_{0}\right)\right)\left[\frac{E_{L_{0},U}\{\exp\left(\beta_{L}^{⊤}w\left(L_{0}\right)+\beta_{U}U\right)\exp\left(-{\int^{\text{​}}}_{0}^{t}\lambda_{0}(s)e^{\beta_{A}^{⊤}v\left(a_{0}\right)+\beta_{L}^{⊤}w\left(L_{0}\right)+\beta_{U}U}ds\right)\}}{E_{L_{0},U}\left\{\exp\left(-{\int^{\text{​}}}_{0}^{t}\lambda_{0}(s)e^{\beta_{A}^{\;}}v\left(a_{0}\right)+\beta_{L}^{1}w\left(L_{0}\right)+\beta_{U}Uds\right)\right\}}\right]$$

The ratio of expectations in the third term in the above expression is a complicated function of both *t* and *a*_0_, and $\lambda_{T^{{\underline{a}}_{0}}}(t)$ no longer takes the Cox model form. A closed form expression for the third term of (17) is not generally available, even in the setting of bivariate normality for *L*_0_, *U*.

Similar results to those provided here for the Cox model were derived by Young and Tchetgen Tchetgen (2014), who focused on a setting in which events are observed in discrete time. In our setting time-dependent treatment status and covariates are observed at discrete time intervals, but events are observed in continuous time. We discuss the results of Young and Tchetgen Tchetgen (2014) further in Section 7.

### 4.4 Summary

When the conditional hazard model $\lambda \left(t|{\bar{A}}_{\left|t\right|},{\bar{L}}_{\left|t\right|},U\right)$ is additive, we have shown that the MSM $\lambda_{T^{{\underline{a}}_{0}}}(t)$ is also additive. The coefficients for ${\bar{a}}_{t}$ in the MSM differ from those in the conditional model except for 0 < *t* < 1—that is, except up to visit *k* = 1. The intercepts in the conditional model differ from those in the MSM at all time points. Even if the conditional hazard model depends on treatment only through the current level, the MSM depends on the whole treatment history.

When the conditional hazardmodel is a Cox model, the MSM is no longer a Coxmodel; instead it takes a complex form with the effect of ${\bar{a}}_{k}$ on the hazard being a complex function of time.

## 5 Simulation Algorithm

It follows from the results of Section 4 that if longitudinal data are simulated according to a conditional additive hazard model, then the marginal hazard model used in a MSM analysis is also additive and hence can be correctly specified. In this section, we describe an example simulation algorithm which results in a known additive form for the MSM. The data generating mechanism corresponds to the DAG in Figure 1, but with event times generated in continuous time. This is intended as a particular illustration of a general approach and the algorithm can easily be modified for other data-generating mechanisms. In Section 6, we illustrate the practical implementation of the algorithm, and R code is provided at https://github.com/ruthkeogh/causal_sim.

Longitudinal data are generated at 5 visits *k* = 0,…,4 for a single time-dependent continuous variable *L*, for example representing a biomarker, and for a binary treatment *A* and continuous variable *U*, representing an individual frailty term. The example algorithm uses a conditional hazard of the form $\lambda \left(t|{\bar{A}}_{\left\lfloor t\right]},{\bar{L}}_{\left\lfloor t\right]},U\right)=\alpha_{0}+\alpha_{A}A_{\left\lfloor t\right]}+\alpha_{L}L_{\left\lfloor t\right\rfloor }+\alpha_{U}U$. Here we focus on constant conditional baseline hazard and constant coefficients, which simplifies the generation of event times. An extension of the algorithm to accommodate more complex forms for the hazard is described in the Supporting Information (Section A4), and is based on generating event times from a piecewise exponential distribution. The conditional hazard at time *t* depends on the current values of *A* and *L*, but not on past values. The implied form of the MSM is $\lambda_{T_{\;}^{{\underline{a}}_{0}}}(t)={\tilde{\alpha }}_{0}(t)+\sum_{j=0}^{\left\lfloor t\right\rfloor }{\tilde{\alpha }}_{Aj}(t)a_{\left\lfloor t\right\rfloor -j}$. In the example algorithm, higher values of the biomarker *L* are associated with higher propensity to receive the treatment and higher hazard. The biomarker value also increases with time. The treatment lowers the value of *L* and lowers the hazard. Event times are generated in the range 0 < *T* < 5 and there is administrative censoring at time 5. Other types of right-censoring could be incorporated, and an example with non-administrative censoring is provided in the example R code.

The steps to generate the longitudinal data are as follows for each individual *i* = 1, …,*n*:

1. Generate the individual frailty term *U* from a normal distribution with mean 0 and standard deviation 0.1.
2. Generate *L*_0_ from a normal distribution with mean *U* and standard deviation 1.
3. Generate *A*_0_ from a Bernoulli distribution with logit Pr(*A*_0_ = 1|*L*_0_) = −2 + 0.5*L*_0_.
4. The conditional hazard is $\lambda \left(t|{\bar{A}}_{\left\lfloor t\right\rfloor },{\bar{L}}_{\left\lfloor t\right\rfloor },U\right)=0.7-0.2A_{\left\lfloor t\right\rfloor }+0.05L_{\left\lfloor t\right\rfloor }+0.05U$. Event times are generated in the period 0 < *t* < 1 as follows. First generate *V* ~ Uniform(0,1) and calculate T* = −log(*V*)/*λ*(*t*|*A*_0_, *L*_0_, *U*). If T* < 1 the event time is set to be *T* = T*. Individuals with T* ≥ 1 remain at risk of the event at time *t* = 1. For individuals who remain at risk of the event at visit time *k* = 1:
5. Generate *L_k_* from a normal distribution with mean 0.8*L*_*k*−1_ − *A*_*k*−1_ + 0.1*k* + *U* and standard deviation 1.
6. Generate *A_k_* from a Bernoulli distribution with logit $\mathrm{Pr}\left(A_{k}=1|{\bar{A}}_{k-1},{\bar{L}}_{k},T\geq k\right)=-2+0.5L_{k}+A_{k-1}$.
7. Generate event times in the period *k* ≤ *t* < *k* + 1. First generate *V* ~ Uniform(0,1) and calculate $T^{*}=-\log(V)/\lambda \left(t|{\bar{A}}_{k},{\bar{L}}_{k},U\right)$. If T* < 1 the event time is set to be *T* = *k* + T*. Individuals with T* ≥ 1 remain at risk of the eventattime *k* + 1.
8. Repeat steps 5–7 for *k* = 2, 3, 4. Individuals who do not have an event time generated in the period 0 < *t* < 5 are administratively censored attime 5.

## 6 Simulation Illustration

### 6.1 Methods and estimands

We illustrate the algorithm described in Section 5 by generating 1000 simulated data sets for each of *n* = 5000 individuals. Our theoretical results imply that the conditional data generating mechanism outlined in Section 5 leads to the correctly specified MSM being of the form $\lambda_{T^{{\underline{a}}_{0}}}(t)={\tilde{\alpha }}_{0}(t)+\sum_{j=0}^{\left\lfloor t\right\rfloor }{\tilde{\alpha }}_{Aj}(t)a_{\lfloor t\rfloor -j}$. This MSM is fitted to each simulated data set using IPTW(MSM-IPTW). Stabilized weights were used for the IPTW estimation and the weights were estimated using logistic regression, with logit $\mathrm{Pr}\left(A_{k}=1|{\bar{A}}_{k-1},T\geq k\right)=\gamma_{0}+\gamma_{A}A_{k-1}$ and logit $\mathrm{Pr}\left(A_{k}=1|{\bar{L}}_{k},{\bar{A}}_{k-1},T\geq k\right)=\gamma_{0}+\gamma_{A}A_{k-1}+\gamma_{L}L_{k}$ (see Supporting Information Section A1). The second model, used in the denominator of the stablised weights, is correctly specified according the data generation mechanism.

The estimands of interest are the cumulative coefficients ${\int^{\text{​}}}_{0}^{t}{\tilde{\alpha }}_{0}(s)ds$ and ${\int^{\text{​}}}_{0}^{t}{\tilde{\alpha }}_{Aj}(s)ds(j=0,1,2,3,4)$ and marginal survival probabilities for two treatment regimes: ‘always treated’ $\mathrm{Pr}(T^{{\underline{a}}_{0}}{\;}^{=1}\geq t))$ and ‘never treated’ $\left(\mathrm{Pr}(T^{{\underline{a}}_{0}}{\;}^{=0}\geq t)\right)$. For each estimandwe present the mean value of the estimates across simulations at times 1, 2, 3, 4, 5 and the corresponding bias. We also obtained the empirical standard errors of the estimates as the standard deviation of the estimates across simulations at times 1, 2, 3, 4, 5. For the bias we obtained Monte Carlo standard errors (Morris et al., 2019). Results are also shown graphically across all time points. Because the analyses are based on a correctly specified MSM and correctly specified models for the weights, we expect the resulting estimates to be approximately unbiased.

### 6.2 Obtaining true values

To calculate the bias we need to know the true values of the estimands. We recommend a simulation-based approach. This involves generating longitudinal data in a similar way to that described in the algorithm but for a large ‘randomized controlled trial’ (RCT) where the relationships between the variables are the same as in the observational study (Figure 1), with the exception that *L_k_* does not affect *A_k_*. Instead, *A_k_* is set by intervention to the fixed value determined by the treatment regime. With 5 visit times and a binary treatment, there are 2^5^ = 32 possible longitudinal treatment regimes. We generated trial data with *m* = 1000 individuals assigned to each of the 32 possible treatment regimes. The 1000 values of *L*_0_ were generated once and set to be the same in each regime. The trial therefore contains in total 32,000 individuals. We simulated 1000 trials. The correctly specified MSM was fitted in each simulated trial data set without any weights—since there is no time-dependent confounding in the trial data there is no need for any weights. This provides estimates of the cumulative coefficients ${\int^{\text{​}}}_{0}^{t}{\tilde{\alpha }}_{0}(s)ds,{\int^{\text{​}}}_{0}^{t}{\tilde{\alpha }}_{Aj}(s)ds,j=0,\ldots ,4$. Estimates of marginal survival probabilities in the ‘always treated’ and ‘never treated’ groups were obtained using (6). Note that the survival probabilities could in fact have been directly estimated using simple proportions from the RCT data, since there is only administrative censoring. This is shown in the example code provided. The true values of the estimands were taken to be the average of the estimates obtained from the large randomized trials across the 1000 simulated data sets.

### 6.3 Results

**Figure 2:**
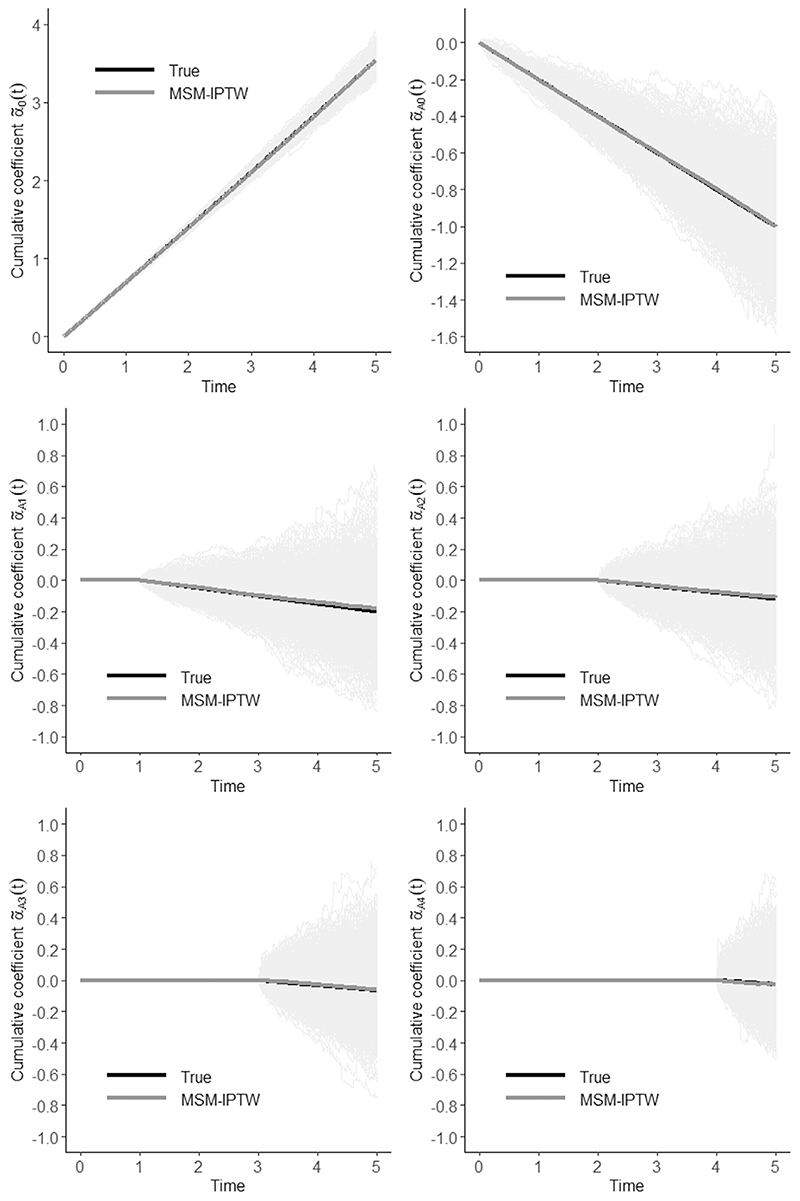
Cumulative coefficients: true values, estimates obtained using MSM-IPTW from 1000 simulated data sets (faded grey lines), and mean estimated cumulative coefficients using MSM-IPTW

**Figure 3:**
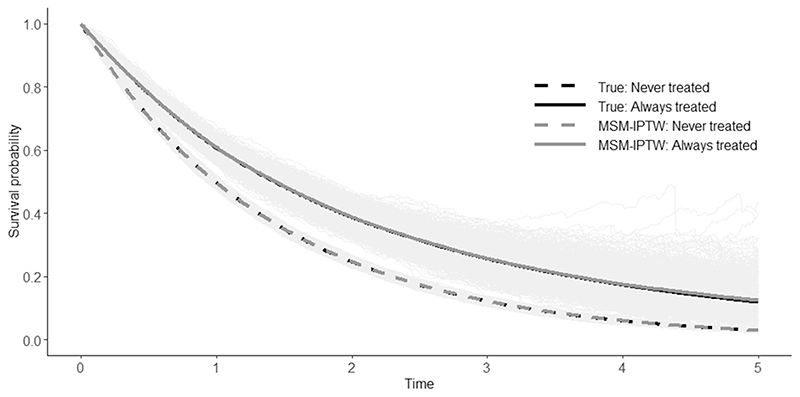
Survival curves for the treatment regimes ‘never treated’ and ‘always treated’: true survival curves, estimated survival curves obtained using MSM-IPTW from 1000 simulated data sets (faded grey lines), and the mean estimated survival curves using MSM-IPTW

The results from the simulation illustration are shown in Tables 1 and 2 and Figures 2 and 3. The estimated cumulative coefficients from the MSM are approximately unbiased. The small bias in some of the cumulative coefficients is thought to be due to finite sample bias, and the plots show that it is negligible. The same applies for the survival probabilities under the ‘always treated’ and ‘never treated’ regimes, which are derived from the cumulative coefficients. The cumulative coefficients are imprecisely estimated, resulting in a large pointwise confidence intervals for the survival curves.

## 7 Discussion

In this paper, we have provided results on the link between the conditional models used in the simulation of longitudinal and time-to-eventdataand the MSMs used in causal inference investigations to estimate the marginal effects of longitudinal treatment regimes on time-to-event outcomes. We have shown (Section 4) that when data are generated under an additive conditional hazard model, the form of the MSM is also additive. By contrast, when data are generated under a conditional Cox model, the form of the MSM is not a Cox model and in fact takes a complex non-standard form. Also, we have described in detail how to simulate longitudinal and time-to-event data based on the additive hazard model (Section 5). We illustrated the simulation algorithm (Section 6), firstly to provide a template for other researchers, and secondly as a validation of the theoretical results in Section 4.2.

Our results and simulation algorithm will help other researchers in the conduct of simulation studies to assess performance of methods under different conditions and to compare properties of different methods. Assessment and comparison of causal inference methods is rarely happening up to now and some comparisons are flawed. Karim et al. (2018) compared results from an analysis using a Cox MSM with an alternative sequential Cox approach described by Gran et al. (2010). However they compared estimands (hazard ratios) from a marginal model with those from a conditional model, concluding incorrectly that the sequential Cox approach provides biased estimates. Gran and Aalen (2019) pointed out that Karim et al. (2018) had not made a fair comparison of the two approaches, firstly because they compared marginal with conditional estimands and secondly because the data generating procedure did not ensure that models were correctly specified under the two approaches.

The results in Section 4 were derived using the g-formula to express the MSM in terms of conditional models for the observed data. As noted in Sections 1 and 3.2, MSMs can be estimated using observed data using IPTW or the g-formula, under the assumptions outlined in Section 3.2. In the simulation illustration in Section 6, we focused on the IPTW approach, which is the most popular (Clare et al., 2018). The general results can also be used to ascertain the form of the correctly specified MSM when using a g-formula analysis with particular specifications for the conditional models. In futurework, itwould be of interest to compare the efficiency of estimates of survival probabilities (for example) obtained using MSMs estimated using IPTW and using the g-formula. Our simulation algorithm could be employed for this purpose, and would enable us to ensure that all models used in the analyses were correctly specified according to the data generating mechanism, including the MSM, the conditional models used in the g-formula analysis, and the propensity score models used in the IPTW analysis.

Our results also highlight the benefits of the additive hazard model for use in causal inference research, which result from its collapsibility property. More causal inference methods are emerging that make use of the additive hazard model for this reason, for example Seaman et al. (2019); Ryalen et al. (2019); Aalen et al. (2019). Ourwork adds to earlier results on how to simulate fromMSMs in the setting of longitudinal and time-to-event data by Havercroft and Didelez (2012), Young et al. (2010), and Young and Tchetgen Tchetgen (2014), who all focused on proportional hazards models. The approach of Havercroft and Didelez (2012) was restricted to a setting similar to that depicted in our DAG in Figure 1, but with the direct arrow from *L_k_* to *Y*_*k*+1_ omitted. This is likely to be unrealistic for most purposes. Also, their algorithm does not generate the data depicted in the DAG in the natural sequential way. Young and Tchetgen Tchetgen (2014) provided results for the Cox model, which are related to those given in Section 4.3, but in a less general situation. They showed that the form of the MSM can be derived under certain conditions. Their results focused on a situation in which the conditional hazard at time *t* depends on $A_{\left\lfloor t\right\rfloor }$, $A_{\lfloor t\rfloor -1}$ and $L_{\left\lfloor t\right\rfloor }$, but not additionally on the further history ${\bar{L}}_{\lfloor t\rfloor -1}$ or ${\bar{A}}_{\lfloor t\rfloor -2}$, and in which the distribution of *L*_*k*+1_ depends on *A_k_* but not on ${\bar{L}}_{k}$ or ${\bar{A}}_{k-1}$. Certain results also required a probit model for the conditional distribution of *L_k_* or the assumption that the event of interest is rare. The earlier work of Young et al. (2010) derived data generating conditions under which a Cox MSM, a structural nested cumulative failure time model (Picciotto et al., 2012) and a structural nested accelerated failure time model (Robins, 1992) can coincide, enabling fair comparison of the three approaches.

While the linear form of the additive hazard model brings advantages, there are also drawbacks. The additive hazard model does not restrict the hazard to be non-negative, which in turn can result in survival probabilities derived from the fitted hazard model being greater than 1. When the hazard depends on continuous covariates it may be impossible to guarantee that the hazard is always positive in simulated data, but model parameter values can be chosen so that a negative hazard is very unlikely. In preliminary investigations for our simulation, we tried different values for the parameters of the hazard model and the parameters determining the distribution of *L_k_*, which affects the hazard, and chose parameter values for the example simulation algorithm and simulation illustration so that the probability of obtaining a negative hazard was negligible. We recommend that researchers using this approach perform similar investigations to ensure that their simulation procedure results in only a small probability of seeing a negative hazard. In the example simulation algorithm, we include a line that sets the hazard to 0 when, as happens with small probability, the hazard is negative.

We focused in this paper on a simplified setting with no loss-to-follow-up except through administrative censoring. The general results apply also when there are other types of right-censoring, including censoring that depends on time-dependent treatment status or covariates, because the compatibility of the MSM with the conditional data generating mechanism is not affected by censoring. When fitting the MSM, censoring that depends on treatment or covariates can be handled through inverse probability of censoring weights, which are multiplied together with the inverse probability of treatment weights. In the R code we provide an example that includes non-administrative censoring.

It is straightforward to extend our simulation algorithm to incorporate more than one *L* variable, and even to more than one treatment variable. We focused on a binary treatment, though the results extend in theory to continuous treatments (e.g. dose). However, estimating MSMs using IPTW is not generally recommended for use with continuous exposures,since it is difficult to specify a correct distribution for the continuous treatment and even mild incorrect specification of the weights model can have significant impact on estimates (Goetgeluk et al., 2008; Naimi et al., 2014). We focused on a setting in which the visits times are regular and the same for all individuals. This is not representative of many of the observational data sets faced in practice, for example from electronic health records. Most causal inference methods for longitudinal and time-to-event data have also focused on this simplified setting. However, recent work has been done to extend to the setting where visits time may be at non-regular intervals and differ across individuals, by Ryalen et al. (2019) who use MSMs based on additive hazard models, and by Seaman et al. (2019), who use structural nested cumulative survival time models. It would be of interest to extend our simulation algorithm to this situation to enable comparisons involving these emerging methods. Finally, we focused on a situation with a single event of interest, such as death. It would also be of interest to extend our results to situations with recurrent events or competing risks following recent developments in this area (Young et al., 2020), therefore enabling simulation investigations to assess causal inference methods available for these more complex scenarios.

## Supplementary Material

Data & Code

Supplementary File

## Figures and Tables

**Table 1:** Cumulative coefficients at times 1–5: true values, mean of the estimates (and empirical SE) obtained using MSM-IPTW from 1000 simulations, and bias in the estimates (and Monte Carlo SE) obtained using MSM-IPTW

Time	True value	MSM-IPTW	
		Mean estimate (Empirical SE)	Bias (Monte Carlo SE)
Cumulative coefficient ${\int^{\text{​}}}_{0}^{t}{\tilde{\alpha }}_{0}(s)ds$			
1	0.700 (0.009)	0.699 (0.016)	−0.001 (0.000)
2	1.408 (0.016)	1.407 (0.028)	−0.000 (0.001)
3	2.128 (0.026)	2.129 (0.045)	0.002 (0.001)
4	2.863 (0.040)	2.867 (0.070)	0.003 (0.002)
5	3.623 (0.058)	3.630 (0.110)	0.007 (0.003)
Cumulative coefficient ${\int^{\text{​}}}_{0}^{t}{\tilde{\alpha }}_{A0}(s)ds$			
1	−0.198 (0.010)	−0.199 (0.037)	−0.001 (0.001)
2	−0.396 (0.017)	−0.397 (0.065)	−0.000 (0.002)
3	−0.594 (0.023)	−0.592 (0.100)	0.001 (0.003)
4	−0.790 (0.033)	−0.788 (0.150)	0.002 (0.005)
5	−0.987 (0.042)	−0.968 (0.231)	0.018 (0.007)
Cumulative coefficient ${\int^{\text{​}}}_{0}^{t}{\tilde{\alpha }}_{A1}(s)ds$ (equal to zero for *t* ≤ 1)			
2	−0.098 (0.013)	−0.102 (0.057)	−0.004 (0.002)
3	−0.195 (0.021)	−0.206 (0.096)	−0.011 (0.003)
4	−0.291 (0.030)	−0.303 (0.155)	−0.013 (0.005)
5	−0.386 (0.039)	−0.390 (0.245)	−0.005 (0.008)
Cumulative coefficient ${\int^{\text{​}}}_{0}^{t}{\tilde{\alpha }}_{A2}(s)ds$(equal to zero for *t* ≤ 2)			
3	−0.077 (0.017)	−0.076 (0.078)	0.001 (0.002)
4	−0.153 (0.027)	−0.153 (0.139)	0.000 (0.004)
5	−0.228 (0.039)	−0.222 (0.232)	0.006 (0.007)
Cumulative coefficient ${\int^{\text{​}}}_{0}^{t}{\tilde{\alpha }}_{A3}(s)ds$(equal to zero for *t* ≤ 3)			
4	−0.060 (0.021)	−0.061 (0.115)	−0.001 (0.004)
5	−0.121 (0.035)	−0.128 (0.211)	−0.007 (0.007)
Cumulative coefficient ${\int^{\text{​}}}_{0}^{t}{\tilde{\alpha }}_{A4}(s)ds$(equal to zero for *t* ≤ 4)			
5	−0.047 (0.028)	−0.039 (0.176)	0.008 (0.006)

**Table 2:** Survival probabilities for the treatment regimes ‘never treated’ and ‘always treated’ at times 1–5: true values, mean of the estimates (and empirical SE) obtained using MSM-IPTW from 1000 simulations, and bias in the estimates (and Monte Carlo SE) obtained using MSM-IPTW

Time	True value	MSM-IPTW	
		Mean estimate (Empirical SE)	Bias (Monte Carlo SE)
Never treated: $\mathrm{Pr}\left(T^{{\underline{a}}_{0}=0}\geq t\right)$			
1	0.497	0.497 (0.008)	0.000 (0.000)
2	0.245	0.245 (0.007)	0.000 (0.000)
3	0.119	0.119(0.005)	−0.000 (0.000)
4	0.057	0.057 (0.004)	−0.000 (0.000)
5	0.027	0.027 (0.003)	−0.000 (0.000)
Always treated: $\mathrm{Pr}\left(T^{{\underline{a}}_{0}=0}\geq t\right)$			
1	0.606	0.607 (0.021)	0.001 (0.001)
2	0.401	0.404 (0.031)	0.003 (0.001)
3	0.283	0.288 (0.040)	0.005 (0.001)
4	0.208	0.216 (0.051)	0.007 (0.002)
5	0.157	0.165 (0.066)	0.009 (0.002)
