# Supplementary material for: Simulating longitudinal data from marginal structural models using the additive hazard model[image]
Source: Biom J. Author manuscript; Available in PMC 2022 Jan 5. (PMC7612178; doi:10.1002/bimj.202000040)

Results that can be reproduced using the R code provided

The README file provides information on the R code that can be used to perform the simulation described in the paper.

For the results presented in the paper the true values for the parameters estimated in the simulation were generated by simulating longitudinal data in a similar way to that described in the simulation algorithm (see section 5 of the paper) but for a large 'randomized controlled trial' where the relationships between the variables are the same as in the observational study (see section 6.2 of the paper). We simulated 1000 trials and the true values of the estimands were taken to be the average of the estimates obtained from the large randomized trials across the 1000 simulated data sets. This takes a few hours to run on a standard laptop (mine, for example) and we used a high performance computer to perform the analyses to generate the results shown in the paper.

To enable readers to try out our the code and obtain results relatively quickly, in the example code we generated the true parameter values using 100 simulated trials, instead of 1000. In the example code we have set the seed so that the results can be reproduced exactly. This document shows the results from running the example code. The table and figure numbers correspond to those in the paper. As one would expect, there are minor differences between the results presented here, and those in the paper.

Table 1: Cumulative coefficients at times $1, \ldots, 5$ at times $1, \ldots, 5$: true values, mean of the estimates (and empirical SE) obtained using MSM-IPTW from 1000 simulations, and bias in the estimates (and Monte Carlo SE) obtained using MSM-IPTW.

		MSM-IPTW	
Time	True value	Mean estimate (Empirical SE)	Bias (Monte Carlo SE)
Cumulative coefficient $\int_0^t \tilde{\alpha}_0(s)ds$			
1	0.698	0.699 (0.015)	0.001 (0.000)
2	1.399	1.400 (0.028)	0.001 (0.001)
3	2.108	2.105 (0.046)	-0.002 (0.001)
4	2.826	2.819 (0.072)	-0.007 (0.002)
5	3.545	3.544 (0.114)	-0.001 (0.004)
Cumulative coefficient $\int_0^t \tilde{\alpha}_{A,0}(s)ds$			
1	-0.199	-0.198 (0.037)	0.001 (0.001)
2	-0.398	-0.400 (0.068)	-0.001 (0.002)
3	-0.599	-0.598 (0.102)	0.000 (0.003)
4	-0.803	-0.800 (0.151)	0.003 (0.005)
5	-1.004	-1.004 (0.219)	0.000 (0.007)
Cumulative coefficient $\int_0^t \tilde{\alpha}_{A,1}(s)ds$ (equal to zero for $t \leq 1$)			
2	-0.052	-0.049 (0.056)	0.003 (0.002)
3	-0.104	-0.097 (0.096)	0.008 (0.003)
4	-0.153	-0.142 (0.156)	0.010 (0.005)
5	-0.200	-0.191 (0.240)	0.009 (0.008)
Cumulative coefficient $\int_0^t \tilde{\alpha}_{A,2}(s)ds$ (equal to zero for $t \leq 2$)			
3	-0.041	-0.041 (0.082)	0.000 (0.003)
4	-0.087	-0.076 (0.150)	0.011 (0.005)
5	-0.125	-0.119 (0.230)	0.006 (0.007)
Cumulative coefficient $\int_0^t \tilde{\alpha}_{A,3}(s)ds$ (equal to zero for $t \leq 3$)			
4	-0.030	-0.028 (0.123)	0.002 (0.004)
5	-0.056	-0.052 (0.219)	0.004 (0.007)
Cumulative coefficient $\int_0^t \tilde{\alpha}_{A,4}(s)ds$ (equal to zero for $t \leq 4$)			
5	-0.027	-0.022 (0.173)	0.006 (0.005)

Figure 2: Cumulative coefficients: true values, estimates obtained using MSM-IPTW from 1000 simulated data sets (faded grey lines), and mean estimated cumulative coefficients using MSM-IPTW.

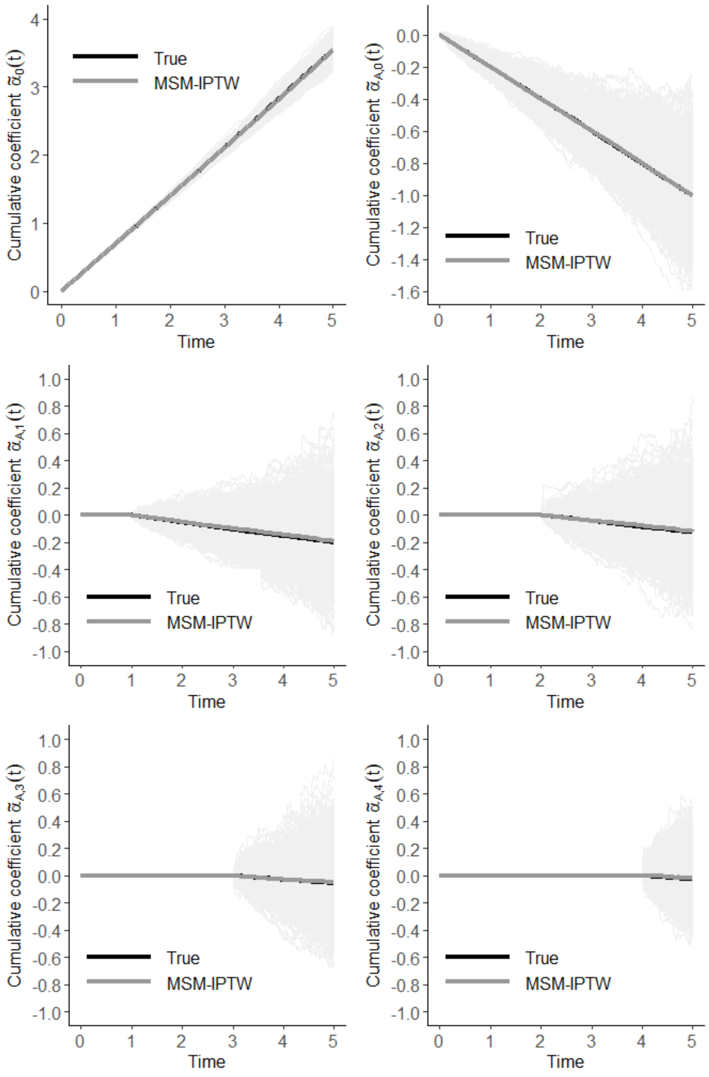

Table 2: Survival probabilities for the treatment regimes 'never treated' and 'always treated' at times $1, \ldots, 5$: true values, mean of the estimates (and empirical SE) obtained using MSM-IPTW from 1000 simulations, and bias in the estimates (and Monte Carlo SE) obtained using MSM-IPTW.

		MSM-IPTW	
Time	True value	Mean estimate (Empirical SE)	Bias (Monte Carlo SE)
Never treated: $\Pr(T^{\underline{a}_0=0} > t)$			
1	0.498	0.497 (0.008)	-0.000 (0.000)
2	0.247	0.247 (0.007)	-0.000 (0.000)
3	0.122	0.122 (0.006)	0.000 (0.000)
4	0.059	0.060 (0.004)	0.001 (0.000)
5	0.029	0.029 (0.003)	0.000 (0.000)
Always treated: $\Pr(T^{\underline{a}_0=1} > t)$			
1	0.607	0.607 (0.020)	-0.000 (0.001)
2	0.387	0.387 (0.030)	0.000 (0.001)
3	0.256	0.257 (0.036)	0.001 (0.001)
4	0.173	0.175 (0.042)	0.002 (0.001)
5	0.119	0.126 (0.053)	0.007 (0.002)

Figure 3: Survival curves for the treatment regimes 'never treated' and 'always treated': true survival curves, estimated survival curves obtained using MSM-IPTW from 1000 simulated data sets (faded grey lines), and the mean estimated survival curves using MSM-IPTW.

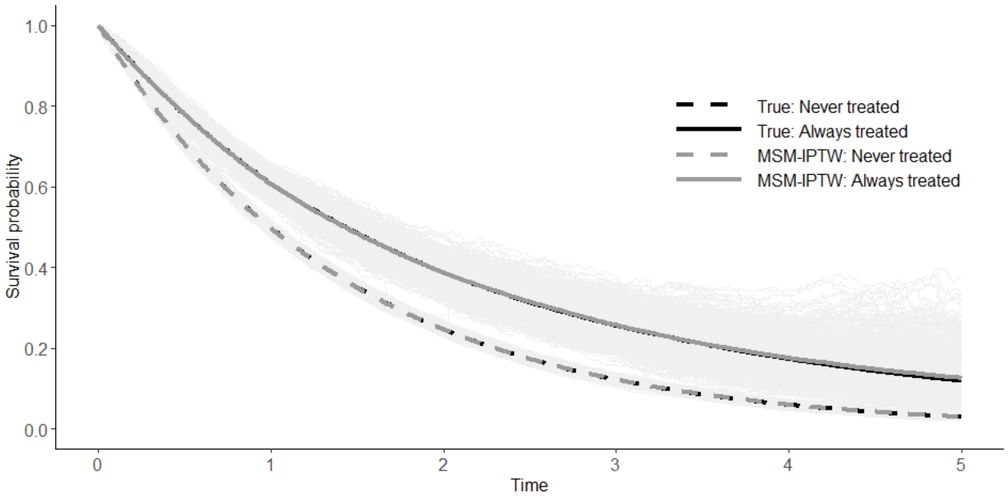

Supplement: Data & Code [file EMS140633-supplement-Data___Code.zip › Code/simulation_replication.pdf]
