# Supplementary figures and images for: Simulating longitudinal data from marginal structural models using the additive hazard model[image]
Source: Biom J. Author manuscript; Available in PMC 2022 Jan 5. (PMC7612178; doi:10.1002/bimj.202000040)

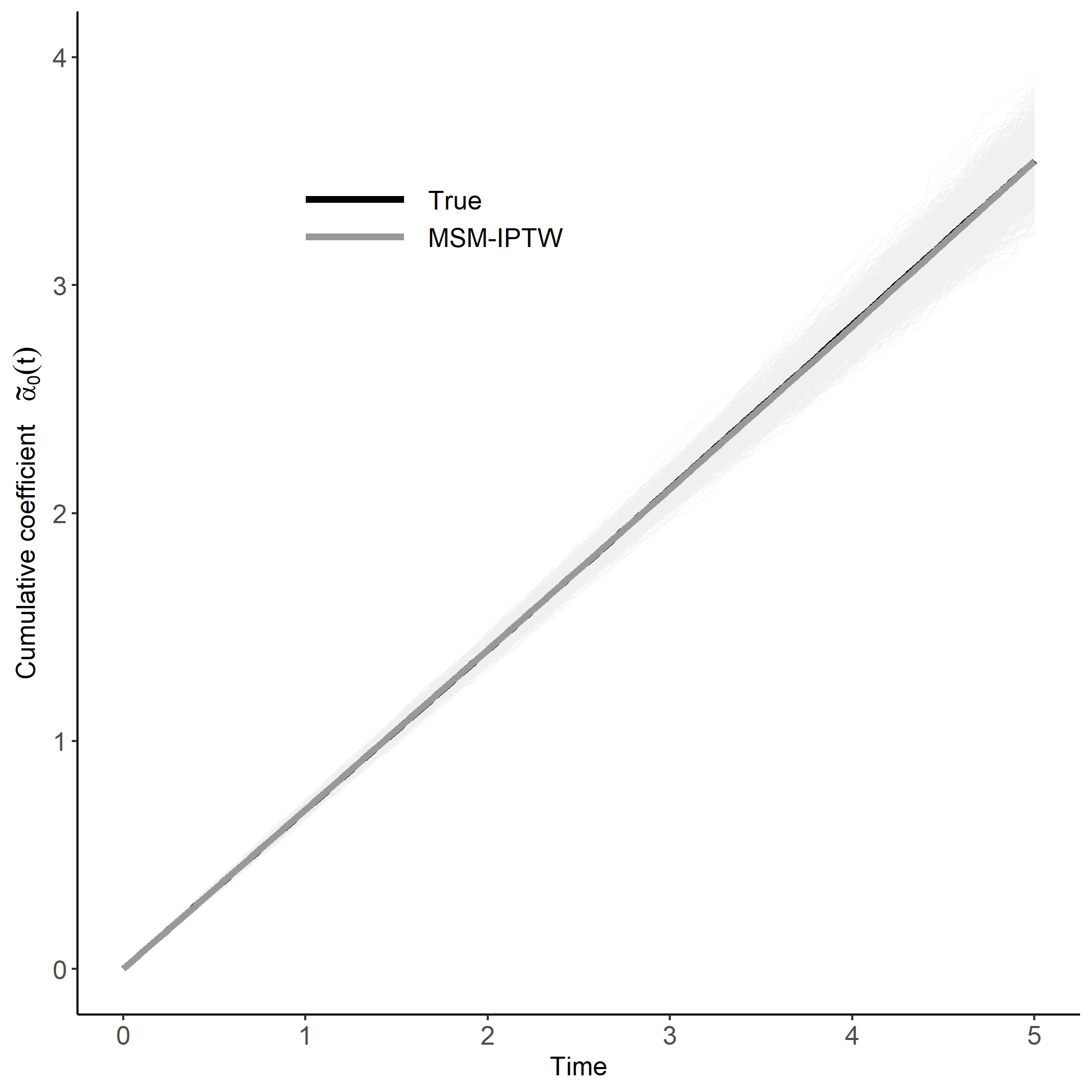

Supplement: Data & Code [file EMS140633-supplement-Data___Code.zip › Code/results/figure2_1.png]

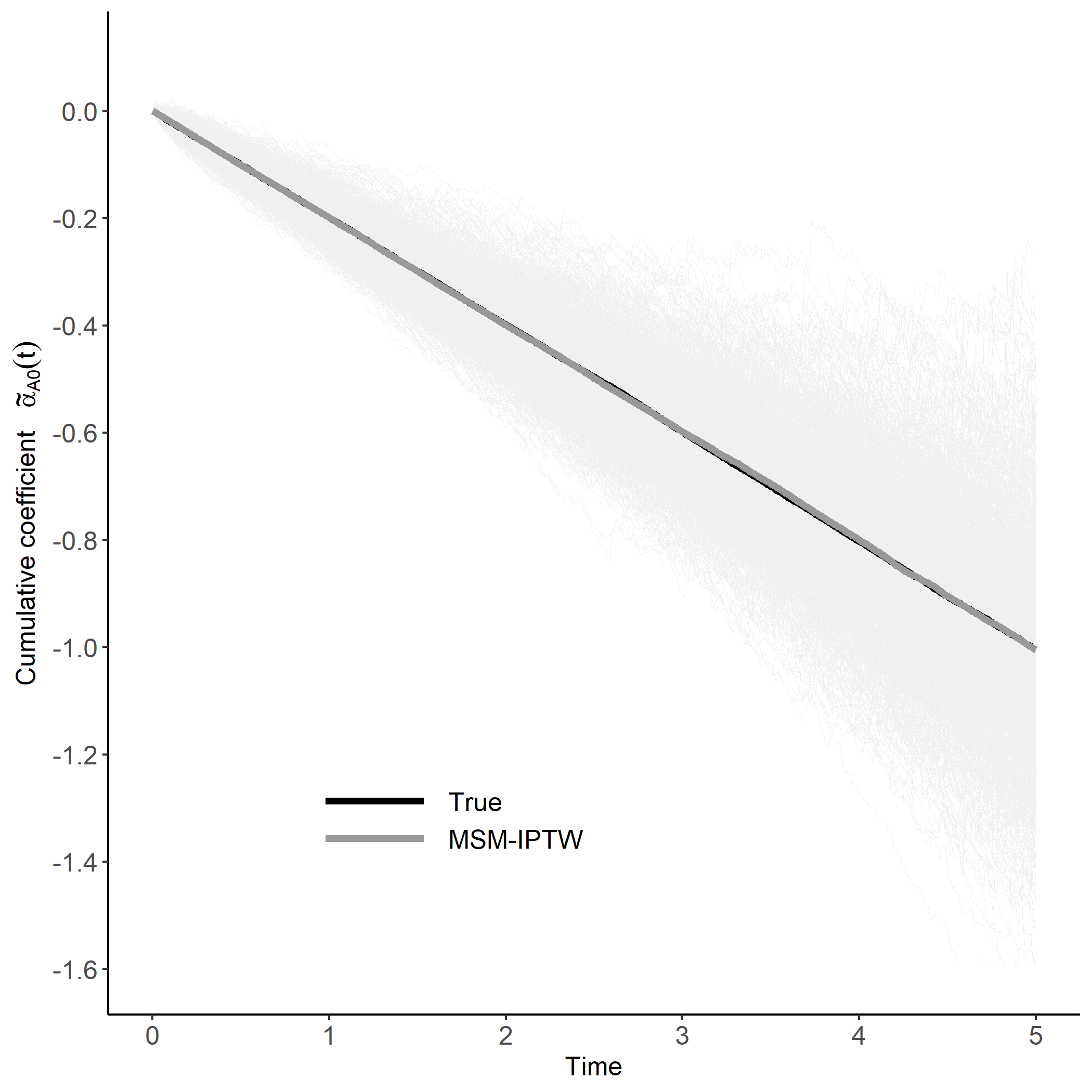

Supplement: Data & Code [file EMS140633-supplement-Data___Code.zip › Code/results/figure2_2.png]

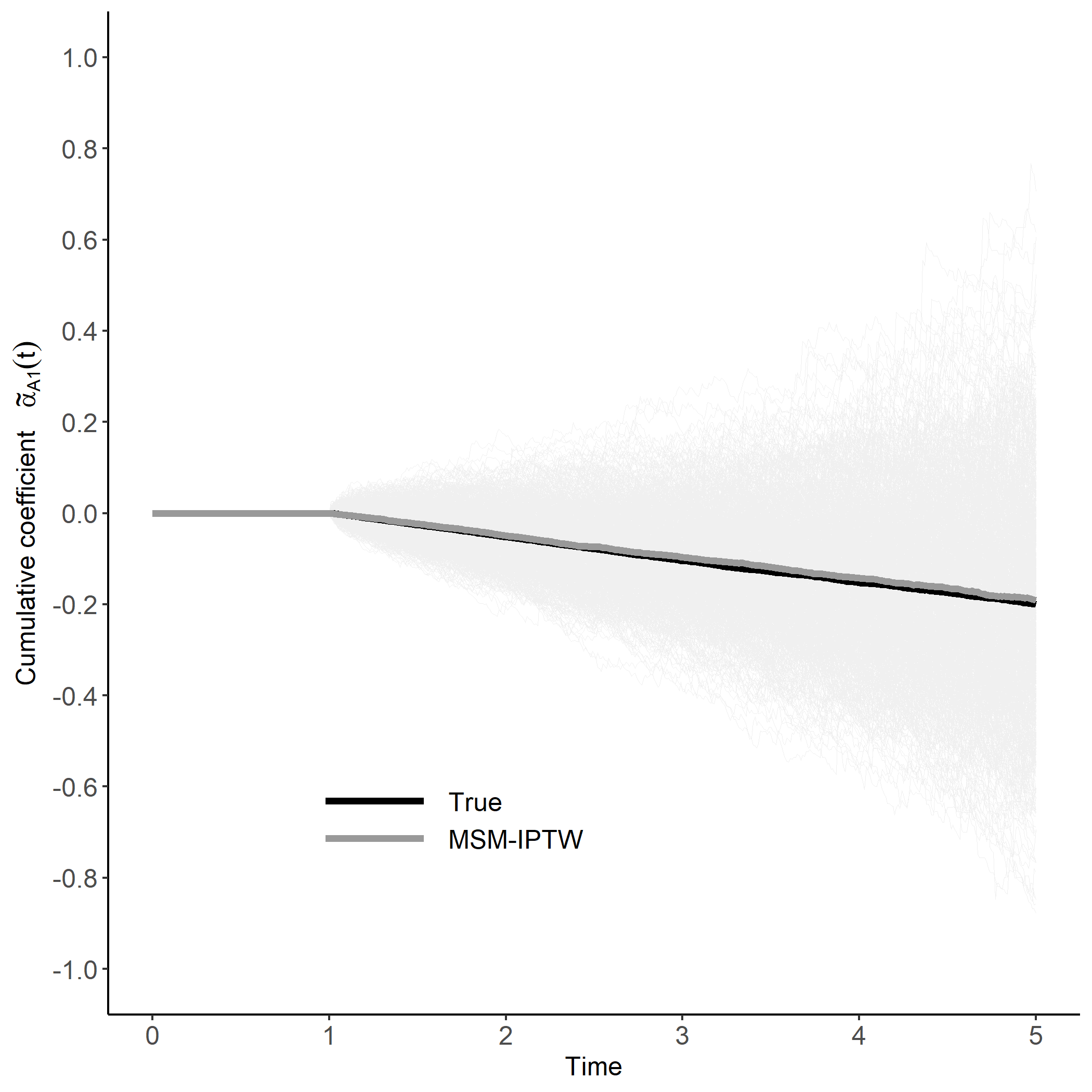

Supplement: Data & Code [file EMS140633-supplement-Data___Code.zip › Code/results/figure2_3.png]

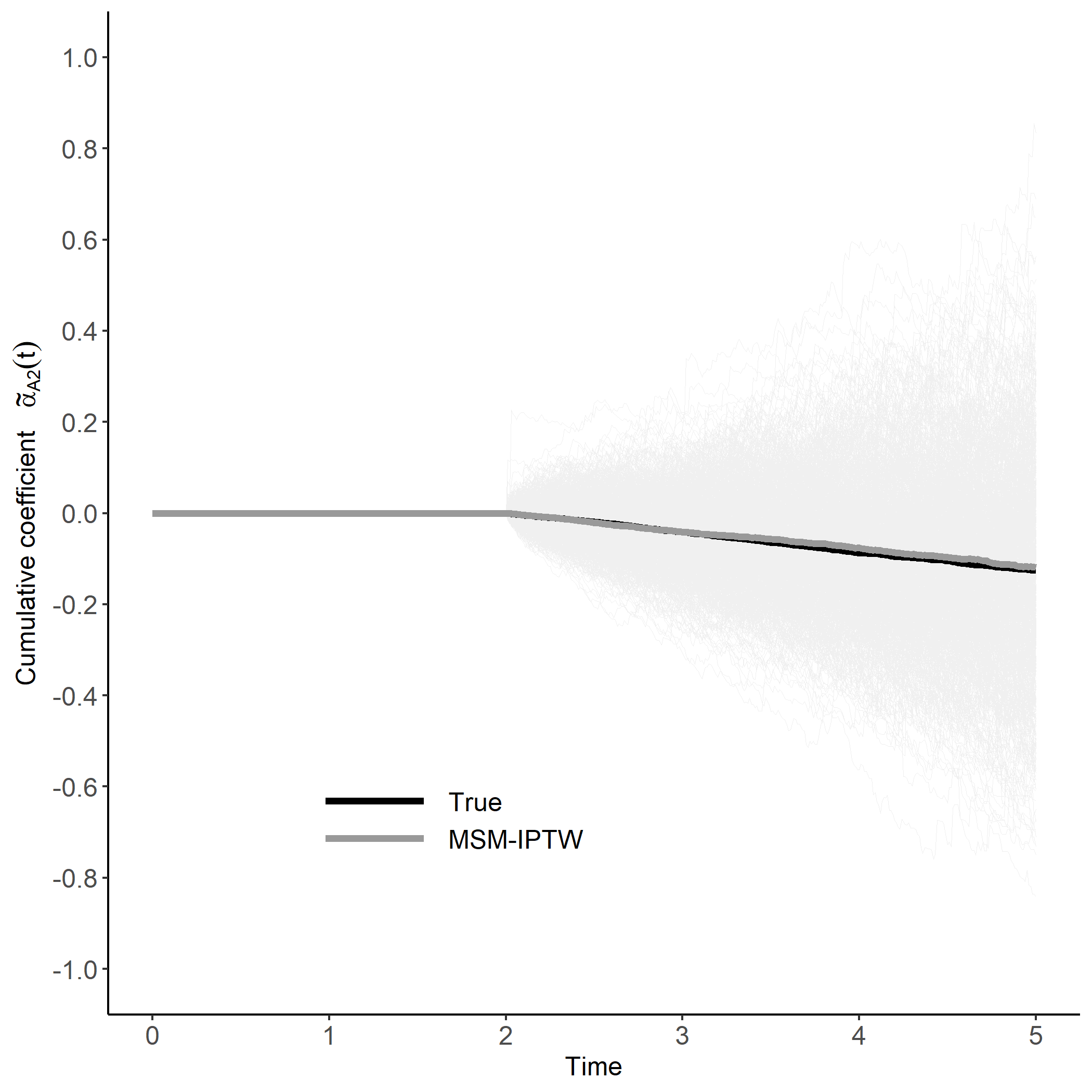

Supplement: Data & Code [file EMS140633-supplement-Data___Code.zip › Code/results/figure2_4.png]

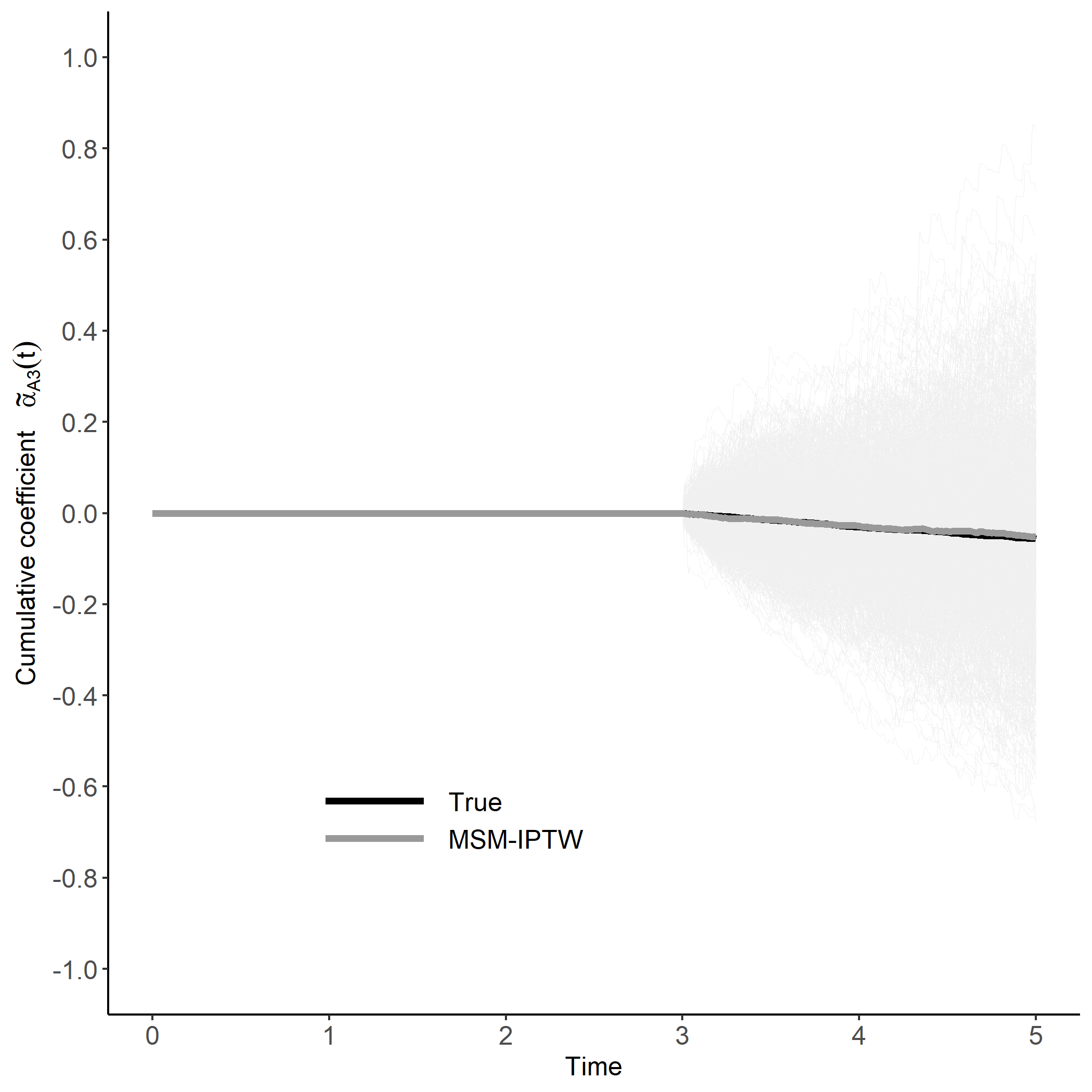

Supplement: Data & Code [file EMS140633-supplement-Data___Code.zip › Code/results/figure2_5.png]

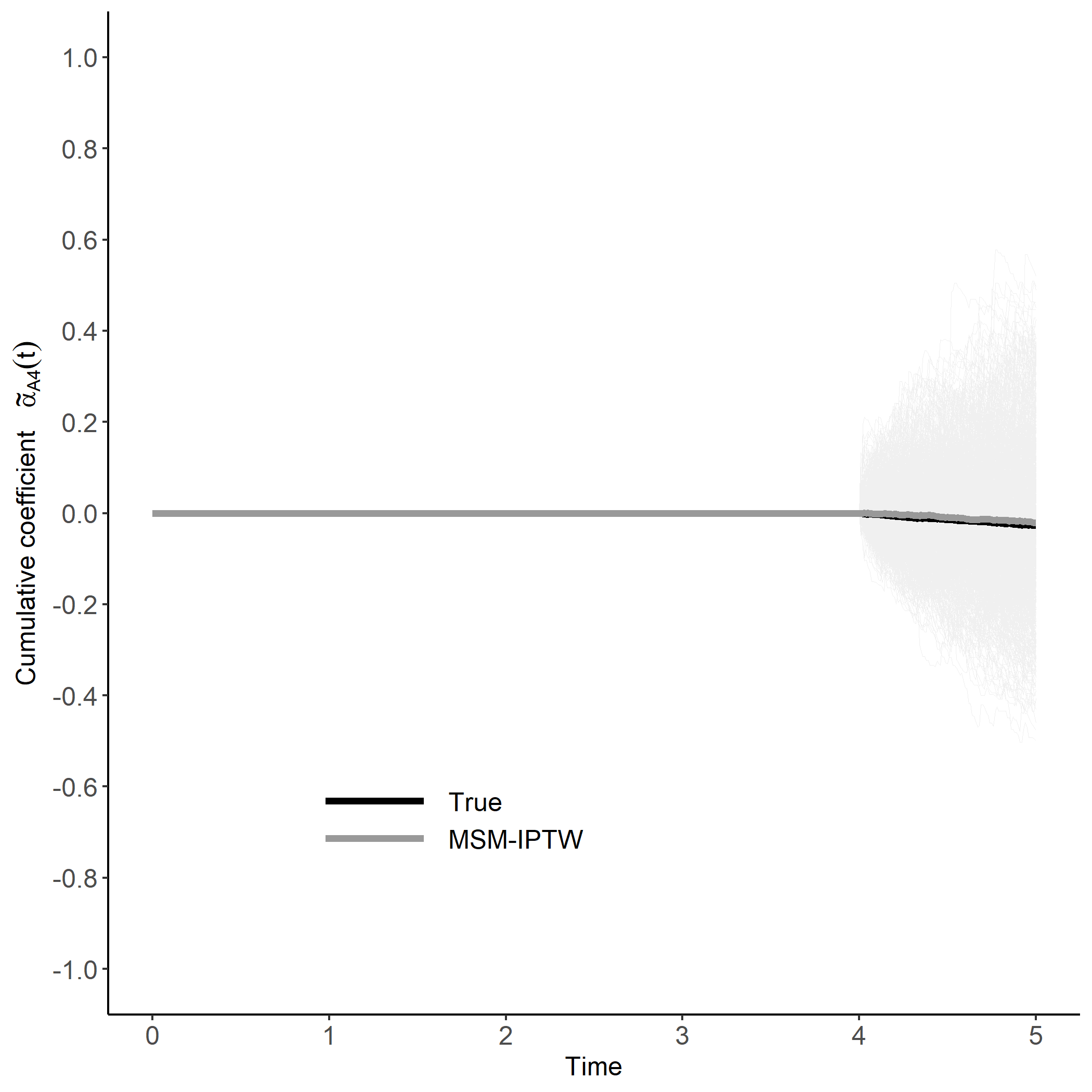

Supplement: Data & Code [file EMS140633-supplement-Data___Code.zip › Code/results/figure2_6.png]

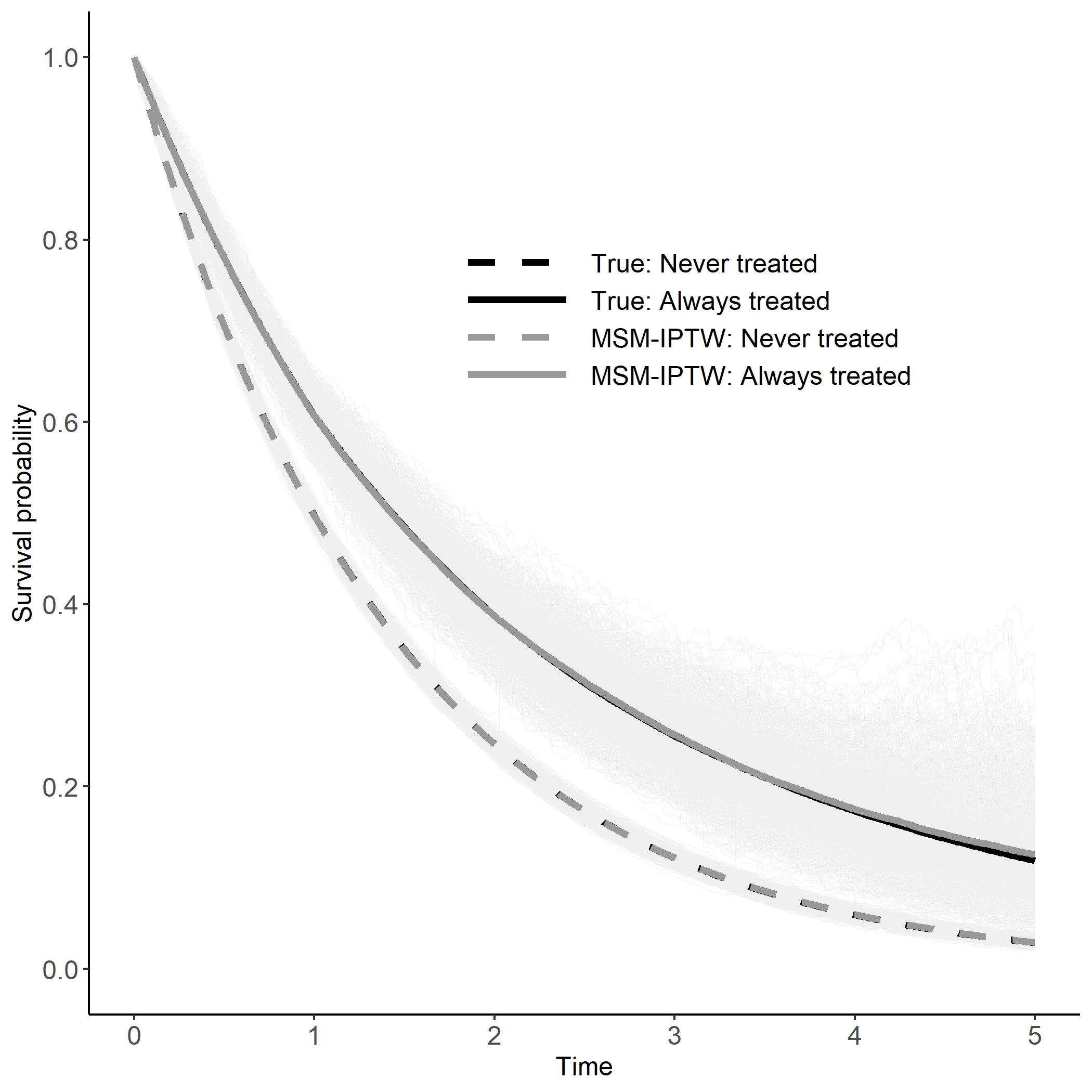

Supplement: Data & Code [file EMS140633-supplement-Data___Code.zip › Code/results/figure3.png]
