# Supplementary material for: Simulating longitudinal data from marginal structural models using the additive hazard model[image]
Source: Biom J. Author manuscript; Available in PMC 2022 Jan 5. (PMC7612178; doi:10.1002/bimj.202000040)
Supplement: Supplementary File [file EMS140633-supplement-Supplementary_File.pdf]

Simulating longitudinal data from marginal structural models using the additive hazard model
Supplementary materials

Ruth H. Keogh[1], Shaun R. Seaman[2], Jon Michael Gran[3], Stijn Vansteelandt[1,4]

[1]Department of Medical Statistics, London School of Hygiene & Tropical Medicine, Keppel Street, London, WC1E 7HT, UK
[2]MRC Biostatistics Unit, University of Cambridge, Institute of Public Health, Forvie Site, Robinson Way, Cambridge CB2 0SR, UK
[3]Oslo Centre for Biostatistics and Epidemiology, Department of Biostatistics, Institute of Basic Medical Sciences, University of Oslo, P.O. Box 1122 Blindern, 0317 Oslo, Norway
[4]Department of Applied Mathematics, Computer Science and Statistics, Ghent University, 9000 Ghent, Belgium

A1 Inverse probability of treatment weights (IPTW)

To estimate MSMs using IPTW, the weight at time t for individual i is the inverse of their probability of their observed treatment pattern up time time t given their time-dependent covariate history (Cole and Hernán, 2008, Daniel et al., 2013)

$$W_i(t) = \prod_{k=0}^{\lfloor t \rfloor} \frac{1}{\Pr(A_k = A_{k,i} | \bar{L}_{k,i}, \bar{A}_{k-1,i}, T \geq k)} \tag{A1}$$

Some individuals can have very large weights, which can results in the parameters of the MSM being estimated very imprecisely, and therefore stabilized weights are typically used. The stabilized weight for individual i is:

$$SW_i(t) = \prod_{k=0}^{\lfloor t \rfloor} \frac{\Pr(A_k = A_{k,i} | \bar{A}_{k-1,i}, T \geq k)}{\Pr(A_k = A_{k,i} | \bar{L}_{k,i}, \bar{A}_{k-1,i}, T \geq k)} \tag{A2}$$

The MSMs in equations (3) and (4) of the main text are marginal over the distribution of the characteristics of the population at time 0. It is also common to condition on baseline characteristics L_0, in which case the MSMs are of the form $\lambda_{T^{\underline{a}_0}}(t|L_0) = \lambda_0(t) \exp\left\{g(\bar{a}_{\lfloor t \rfloor}, L_0; \beta)\right\}$ and $\lambda_{T^{\underline{a}_0}}(t|L_0) = \alpha_0(t) + g(\bar{a}_{\lfloor t \rfloor}, L_0; \alpha(t))$. The contributions of L_0 may be through main effects only, or there may be interactions between L_0 and $\bar{a}_{\lfloor t \rfloor}$. When the MSM is conditional on L_0, the numerator in the stabilized weights may also condition on L_0, and vice-versa:

$$SW_i(t) = \prod_{k=0}^{\lfloor t \rfloor} \frac{\Pr(A_k = A_{k,i} | \bar{A}_{k-1,i}, L_{0,i}, T \geq k)}{\Pr(A_k = A_{k,i} | \bar{L}_{k,i}, \bar{A}_{k-1,i}, T \geq k)} \tag{A3}$$

A2 MSMs using conditional additive hazard models: additional results

In this section we use the general results from Section 4.2 to derive the form of the MSM $\lambda_{T^{\underline{a}_0}}(t)$ when the conditional additive hazard is of the form

$$\lambda(t|\bar{A}_{\lfloor t\rfloor},\bar{L}_{\lfloor t\rfloor},U)=\alpha_0(t)+\sum_{j=0}^{\lfloor t\rfloor}\alpha_{Aj}(t)A_{\lfloor t\rfloor-j}+\sum_{j=0}^{\lfloor t\rfloor}\alpha_{Lj}(t)L_{\lfloor t\rfloor-j}+\alpha_U(t)U \tag{A4}$$

and when the covariates are normally and conditionally normally distributed as follows

$$U\sim N(\nu,\phi^2)$$

$$L_0|U\sim N(\theta_{00}+\theta_{0U}U,\sigma_0^2)$$

$$L_1|A_0=a_0,L_0,U,T\geq 1\sim N(\theta_{10}+\theta_{1A}a_0+\theta_{1L}L_0+\theta_{1U}U,\sigma_1^2)$$

We use the following notation for the cumulative coefficients of the conditional additive hazard model

$$\mathcal{A}_U=\int_0^t\alpha_U(s)ds,$$

$$\mathcal{A}_{L0}=\int_0^t\alpha_{L0}(s)ds,\quad \mathcal{A}_{L0,1}=\int_1^t\alpha_{L0}(s)ds$$

$$\mathcal{A}_{L1}=\int_0^t\alpha_{L1}(s)ds$$

The results given below use the general results that for $X\sim N(\mu,\sigma^2)$

$$E\left\{\exp(-Xw)\right\}=\exp\left(-\mu w+\sigma^2w^2/2\right) \tag{A5}$$

$$E\left\{X\exp(-Xw)\right\}=-\frac{d}{dw}E\left\{\exp(-Xw)\right\}=(\mu-\sigma^2w)\exp\left(-\mu w+\sigma^2w^2/2\right) \tag{A6}$$

For $0<t<1$ the conditional hazard in (A4) is $\lambda(t|A_0,L_0,U)=\alpha_0(t)+\alpha_{A0}(t)A_0+\alpha_{L0}(t)L_0+\alpha_U(t)U$. Using the result in (15) in the main text, the form of $\lambda_{T^{\underline{a}_0}}(t)$ for $0<t<1$ is

$$\begin{aligned}\lambda_{T^{\underline{a}_0}}(t)&=\alpha_0(t)+\alpha_{A0}(t)a_0+\frac{E_{L_0,U}\left\{(\alpha_{L0}(t)L_0+\alpha_U(t)U)\exp\left(-\mathcal{A}_{L0}L_0-\mathcal{A}_UU\right)\right\}}{E_{L_0,U}\left\{\exp\left(-\mathcal{A}_{L0}L_0-\mathcal{A}_UU\right)\right\}}\\&=\alpha_0(t)+\alpha_{A0}(t)a_0\\&\quad+\frac{E_U\left\{\exp\left(-\mathcal{A}_UU\right)\left[\alpha_{L0}(t)E_{L_0|U}\left\{L_0\exp\left(-\mathcal{A}_{L0}L_0\right)\right\}+\alpha_U(t)UE_{L_0|U}\left\{\exp\left(-\mathcal{A}_{L0}L_0\right)\right\}\right]\right\}}{E_U\left[\exp\left(-\mathcal{A}_UU\right)E_{L_0|U}\left\{\exp\left(-\mathcal{A}_{L0}L_0\right)\right\}\right]}\end{aligned} \tag{A7}$$

We let

$$C=\exp\left(-\theta_{00}\mathcal{A}_{L0}+\sigma_0^2\mathcal{A}_{L0}^2/2\right)$$

$$D=\exp\left\{-\nu(\theta_{0U}\mathcal{A}_{L0}+\mathcal{A}_U)+\phi^2(\theta_{0U}\mathcal{A}_{L0}+\mathcal{A}_U)^2/2\right\}$$

Under the assumed normal distributions for U and $L_0|U$ and using the results in (A5) and (A6) it can be shown that

$$\begin{aligned}
E_{L_0|U}\left\{\exp\left(-\mathcal{A}_{L0}L_0\right)\right\} &= C\exp\left(-\theta_{0U}\mathcal{A}_{L0}U\right)\\
E_{L_0|U}\left\{L_0\exp\left(-\mathcal{A}_{L0}L_0\right)\right\} &= \left(\theta_{00}+\theta_{0U}U-\sigma_0^2\mathcal{A}_{L0}\right)C\exp\left(-\theta_{0U}\mathcal{A}_{L0}U\right)\\
E_U\left[\exp\left(-\mathcal{A}_U U\right)E_{L_0|U}\left\{\exp\left(-\mathcal{A}_{L0}L_0\right)\right\}\right] &= CD\\
E_U\left[U\exp\left(-\mathcal{A}_U U\right)E_{L_0|U}\left\{\exp\left(-\mathcal{A}_{L0}L_0\right)\right\}\right] &= CD\left(\nu-\phi^2\mathcal{A}_U-\phi^2\theta_{0U}\mathcal{A}_{L0}\right)\\
E_U\left[\exp\left(-\mathcal{A}_U U\right)E_{L_0|U}\left\{L_0\exp\left(-\mathcal{A}_{L0}L_0\right)\right\}\right] &= CD\left\{\theta_{00}-\sigma_0^2\mathcal{A}_{L0}+\theta_{0U}\left(\nu-\phi^2\mathcal{A}_U-\phi^2\theta_{0U}\mathcal{A}_{L0}\right)\right\}
\end{aligned}$$

It follows that $\lambda_{T^{\underline{a}_0}}(t)$ for $0 < t < 1$ in (A7) can be written

$$\lambda_{T^{\underline{a}_0}}(t) = \tilde{\alpha}_0(t) + \alpha_{A0}(t)a_0 \tag{A8}$$

where

$$\tilde{\alpha}_0(t) = \alpha_0(t) + \alpha_{L0}(t)\left(\theta_{00}-\sigma_0^2\mathcal{A}_{L0}\right) + \left(\alpha_{L0}(t)\theta_{0U}+\alpha_U(t)\right)\left(\nu-\phi^2\mathcal{A}_U-\phi^2\theta_{0U}\mathcal{A}_{L0}\right) \tag{A9}$$

For $1 \le t < 2$ the conditional hazard in (A4) is $\lambda(t|\bar{A}_1,\bar{L}_1,U) = \alpha_0(t)+\alpha_{A0}(t)A_1+\alpha_{A1}(t)A_0+\alpha_{L0}(t)L_1+\alpha_{L1}(t)L_0+\alpha_U(t)U$. Using the result in (16) in the main text, the form of $\lambda_{T^{\underline{a}_0}}(t)$ for $1 \le t < 2$ is

$$\begin{aligned}
&\lambda_{T^{\underline{a}_0}}(t) = \alpha_0(t)+\alpha_{A0}(t)a_1+\alpha_{A1}(t)a_0+\\
&\quad\frac{E_{L_0,U}\left[E_{L_1|A_0=a_0,L_0,U,T\ge 1}\left\{\left(\alpha_{L0}(t)L_1+\alpha_{L1}(t)L_0+\alpha_U(t)U\right)\exp\left(-\mathcal{A}_{L0,1}L_1-\mathcal{A}_{L1}L_0-\mathcal{A}_U U\right)\right\}\right]}{E_{L_0,U}\left[E_{L_1|A_0=a_0,L_0,U,T\ge 1}\left\{\exp\left(-\mathcal{A}_{L0,1}L_1-\mathcal{A}_{L1}L_0-\mathcal{A}_U U\right)\right\}\right]}\\
&\quad=\alpha_0(t)+\alpha_{A0}(t)a_1+\alpha_{A1}(t)a_0+\\
&\quad\frac{E_U\left\{\exp\left(-\mathcal{A}_U U\right)E_{L_0|U}\left[\exp\left(-\mathcal{A}_{L1}L_0\right)E_{L_1|A_0=a_0,L_0,U,T\ge 1}\left\{\left(\alpha_{L0}(t)L_1+\alpha_{L1}(t)L_0+\alpha_U(t)U\right)\exp\left(-\mathcal{A}_{L0,1}L_1\right)\right\}\right]\right\}}{E_U\left\{\exp\left(-\mathcal{A}_U U\right)E_{L_0|U}\left[\exp\left(-\mathcal{A}_{L1}L_0\right)E_{L_1|A_0=a_0,L_0,U,T\ge 1}\left\{\exp\left(-\mathcal{A}_{L0,1}L_1\right)\right\}\right]\right\}}
\end{aligned} \tag{A10}$$

We let

$$\begin{aligned}
F &= \exp\left\{-(\theta_{10}+\theta_{1A}a_0)\mathcal{A}_{L0,1}+\sigma_1^2\mathcal{A}_{L0,1}^2/2\right\}\\
G &= \exp\left\{-\theta_{00}\left(\mathcal{A}_{L1}+\theta_{1L}\mathcal{A}_{L0,1}\right)+\sigma_0^2\left(\mathcal{A}_{L1}+\theta_{1L}\mathcal{A}_{L0,1}\right)^2/2\right\}\\
H &= \exp\left\{-\nu\left(\mathcal{A}_U+\theta_{1U}\mathcal{A}_{L0,1}+\theta_{0U}\mathcal{A}_{L1}+\theta_{0U}\theta_{1L}\mathcal{A}_{L0,1}\right)\right.\\
&\qquad\left.+\phi^2\left(\mathcal{A}_U+\theta_{1U}\mathcal{A}_{L0,1}+\theta_{0U}\mathcal{A}_{L1}+\theta_{0U}\theta_{1L}\mathcal{A}_{L0,1}\right)^2/2\right\}\\
J &= \alpha_{L0}(t)\left(\theta_{10}-\sigma_1^2\mathcal{A}_{L0,1}\right)+\left(\alpha_{L1}(t)+\alpha_{L0}(t)\theta_{1L}\right)\left\{\theta_{00}-\sigma_0^2\left(\theta_{1L}\mathcal{A}_{L0,1}+\mathcal{A}_{L1}\right)\right\}\\
K &= \alpha_U(t)+\alpha_{L0}(t)\theta_{1U}+\theta_{0U}\left(\alpha_{L1}(t)+\alpha_{L0}(t)\theta_{1L}\right)
\end{aligned}$$

Under the assumed normal distributions for U, $L_0|U$, $L_1|A_0 = a_0, L_0, U, T \ge 1$ and using the results in (A5) and (A6), the term in the denominator of the ratio of expectations in the third term of (A10) can be derived sequentially as follows:

$$\begin{aligned}
E_{L_1|A_0=a_0,L_0,U,T\ge 1}\left\{\exp\left(-\mathcal{A}_{L0,1}L_1\right)\right\} &= F\exp\left(-\theta_{1L}\mathcal{A}_{L0,1}L_0-\theta_{1U}\mathcal{A}_{L0,1}U\right)\\
E_{L_0|U}\left[\exp\left(-\mathcal{A}_{L1}L_0\right)E_{L_1|A_0=a_0,L_0,U,T\ge 1}\left\{\exp\left(-\mathcal{A}_{L0,1}L_1\right)\right\}\right] &= FG\times\\
&\quad\exp\left\{-\left(\theta_{1U}\mathcal{A}_{L0,1}+\theta_{0U}\theta_{1L}\mathcal{A}_{L0,1}+\theta_{0U}\mathcal{A}_{L1}\right)U\right\}
\end{aligned}$$

$$E_U\left\{\exp\left(-\mathcal{A}_U U\right)E_{L_0|U}\left[\exp\left(-\mathcal{A}_{L1}L_0\right)E_{L_1|A_0=a_0,L_0,U,T\ge 1}\left\{\exp\left(-\mathcal{A}_{L0,1}L_1\right)\right\}\right]\right\} = FGH$$

Similarly, the terms in the numerator of the ratio of expectations in the third term of (A10) can be derived sequentially as follows:

$$E_{L_1|A_0=a_0,L_0,U,T\geq 1}\left\{(\alpha_{L0}(t)L_1+\alpha_{L1}(t)L_0+\alpha_U(t)U)\exp\left(-\mathcal{A}_{L0,1}L_1\right)\right\} = \exp\left(-\theta_{1L}\mathcal{A}_{L0,1}L_0-\theta_{1U}\mathcal{A}_{L0,1}U\right)\times$$
$$F\left\{\alpha_{L0}(t)\left(\theta_{10}+\theta_{1A}a_0-\sigma_1^2\mathcal{A}_{L0,1}\right)+\left(\alpha_{L1}(t)+\alpha_{L0}(t)\theta_{1L}\right)L_0+\left(\alpha_U(t)+\alpha_{L0}(t)\theta_{1U}\right)U\right\}$$

$$E_{L_0|U}\left[\exp\left(-\mathcal{A}_{L1}L_0\right)E_{L_1|A_0=a_0,L_0,U,T\geq 1}\left\{(\alpha_{L0}(t)L_1+\alpha_{L1}(t)L_0+\alpha_U(t)U)\exp\left(-\mathcal{A}_{L0,1}L_1\right)\right\}\right] = FG\left(J+\alpha_{L0}(t)\theta_{1A}a_0+KU\right)\exp\left\{-\left(\theta_{0U}\theta_{1L}\mathcal{A}_{L0,1}+\theta_{0U}\mathcal{A}_{L1}+\theta_{1U}\mathcal{A}_{L0,1}\right)U\right\}$$

$$E_U\left\{\exp\left(-\mathcal{A}_UU\right)E_{L_0|U}\left[\exp\left(-\mathcal{A}_{L1}L_0\right)E_{L_1|A_0=a_0,L_0,U,T\geq 1}\left\{(\alpha_{L0}(t)L_1+\alpha_{L1}(t)L_0+\alpha_U(t)U)\exp\left(-\mathcal{A}_{L0,1}L_1\right)\right\}\right]\right\} = FGH\left[J+\alpha_{L0}(t)\theta_{1A}a_0+K\left\{\nu-\phi^2\left(\mathcal{A}_U+\theta_{1U}\mathcal{A}_{L0,1}+\theta_{0U}\mathcal{A}_{L1}+\theta_{0U}\theta_{1L}\mathcal{A}_{L0,1}\right)\right\}\right]$$

It can be shown using the above results that

$$\lambda_{T^{\underline{a}_0}}(t)=\tilde{\alpha}_0(t)+\alpha_{A0}(t)a_1+\tilde{\alpha}_{A1}(t)a_0 \tag{A11}$$

where

$$\tilde{\alpha}_0(t)=\alpha_0(t)+K\left\{\nu-\phi^2\left(\mathcal{A}_U+\theta_{1U}\mathcal{A}_{L0,1}+\theta_{0U}\mathcal{A}_{L1}+\theta_{0U}\theta_{1L}\mathcal{A}_{L0,1}\right)\right\}+J$$

and

$$\tilde{\alpha}_{A1}(t)=\alpha_{A1}(t)+\alpha_{L0}(t)\theta_{1A}$$

We have therefore derived the form of the MSM $\lambda_{T^{\underline{a}_0}}(t)$ for $0<t<1$ and $1\leq t<2$ when the conditional hazard is of the form in (A4) and when the covariates are normally and conditionally normally distributed. As shown in more general results in Section 4.2 of the main text, the MSMs have an additive form. However, the above results show that the formulae for the coefficients in the MSM take quite a complicated form even in this relatively simple setting. The expressions would become further complicated if there were multiple time-dependent covariates L and when the conditional distributions for the covariates given the past were not normal, in which case there will not in general exist closed form expressions for the coefficients of the MSM. In Section 6.2 of the main text we outline a simulation-based procedure for obtaining the true values of the coefficients in the MSM.

A3 Incorporating interactions

In Section 4.2 of the main text, we considered the conditional additive hazard model given in equation (14). Suppose instead that there was also an interaction between $\bar{A}_t$ and $\bar{L}_t$:

$$\lambda(t|\bar{A}_{\lfloor t\rfloor},\bar{L}_{\lfloor t\rfloor},U)=\alpha_0(t)+\alpha_A^\top(t)v(\bar{A}_t)+\alpha_L^\top(t)w(\bar{L}_t)+\alpha_{AL}^\top(t)q(\bar{A}_t,\bar{L}_t)+\alpha_U(t)U \tag{A12}$$

where $q(\bar{A}_{\lfloor t\rfloor},\bar{L}_{\lfloor t\rfloor})$ denotes a vector values function of interactions between $\bar{A}_t$ and $\bar{L}_t$. Following the same workings as in Section 4.2 of the main text, it can be shown that for $0<t<1$

$$\lambda_{T^{\underline{a}_0}}(t)=\alpha_0(t)+\alpha_A^\top(t)v(a_0)+\frac{E_{L_0,U}\left\{\left(\alpha_L^\top(t)w(L_0)+\alpha_{AL}^\top(t)q(a_0,L_0)+\alpha_U(t)U\right)r_0(t)\right\}}{E_{L_0,U}\left\{r_0(t)\right\}} \tag{A13}$$

where $r_0(t)=\exp\Big(-\int_0^t(\alpha_L^\top(s)w(L_0)+\alpha_{AL}^\top(s)q(a_0,L_0)+\alpha_U(s)U)ds\Big)$.

For $1 \leq t < 2$ we have

$$\lambda_{T^{\underline{a}_0}}(t) = \alpha_0(t) + \alpha_A^\top(t)v(\bar{a}_1) + \frac{E_{L_0,U}\left[E_{L_1|A_0=a_0,L_0,U,T\geq 1}\left\{\left(\alpha_L^\top(t)w(\bar{L}_1) + \alpha_{AL}^\top(t)q(\bar{a}_1,\bar{L}_1) + \alpha_U(t)U\right)r_1(t)\right\}\right]}{E_{L_0,U}\left[E_{L_1|A_0=a_0,L_0,U,T\geq 1}\left\{r_1(t)\right\}\right]} \tag{A14}$$

where $r_1(t) = r_0(t)\exp\left\{-\int_1^t\left(\alpha_L^\top(s)f(\bar{L}_1) + \alpha_{AL}^\top(s)q(\bar{a}_1,\bar{L}_1) + \alpha_U(s)U\right)ds\right\}$.

For $1 \leq t < 2$ the intercept and the coefficients for a_0 and a_1 in the MSM are different from those in the conditional model. The MSM also involves an interaction between a_0 and a_1 even if there is no interaction between a_0 and a_1 in the conditional hazard model.

A4 Simulation algorithm: extensions

In section 5 of the main text we described a simulation algorithm for longitudinal and time-to-event data, using a conditional additive hazard model of the form $\lambda(t|\bar{A}_{\lfloor t\rfloor}, \bar{L}_{\lfloor t\rfloor}, U) = \alpha_0 + \alpha_A A_{\lfloor t\rfloor} + \alpha_L L_{\lfloor t\rfloor} + \alpha_U U$. The algorithm can be extended to accommodate a more general form for the conditional hazard including time-varying coefficients: $\lambda(t|\bar{A}_{\lfloor t\rfloor}, \bar{L}_{\lfloor t\rfloor}, U) = \alpha_0(t) + \alpha_A^\top(t)v(\bar{A}_{\lfloor t\rfloor}) + \alpha_L^\top(t)w(\bar{L}_{\lfloor t\rfloor}) + \alpha_U(t)U$. For the simulation the investigator needs to specify the functional forms for the coefficients. One way to simulate data in this more general setting is by generating event times using a piecewise exponential distribution, as we outline below. Further extensions to include additional terms such as interaction terms follow directly.

A general form for the simulation algorithm is as follows:

1. Generate the individual frailty term U.
2. Generate L_0 conditional on U.
3. Generate A_0 from a Bernoulli distribution conditional on L_0.
4. The conditional hazard is $\lambda(t|\bar{A}_{\lfloor t\rfloor}, \bar{L}_{\lfloor t\rfloor}, U) = \alpha_0(t) + \alpha_A^\top(t)v(\bar{A}_{\lfloor t\rfloor}) + \alpha_L^\top(t)w(\bar{L}_{\lfloor t\rfloor}) + \alpha_U(t)U$. Event times are generated in the period $0 < t < 1$ using a piecewise exponential distribution on a grid from 0 to 1 in increments of length 0.1 (this could be made smaller or larger). The procedure is as follows. First generate $V \sim \text{Uniform}(0,1)$ and calculate $T^* = -\log(V)/\lambda(0|A_0, L_0, U)$. If $T^* < 0.1$ the event time is set to be $T = T^*$. If $T^* \geq 0.1$, then for $w = 0.1, 0.2, \ldots, 0.9$:
   (i) Generate $v \sim \text{Uniform}(0,1)$ and calculate $T^* = -\log(V)/\lambda(w|A_0, L_0, U)$.
   (ii) If $T^* < 0.1$ the event time is set to be $T = w + T^*$.
   (iii) If $T^* \geq 0.1$ move to the next value of w and return to (i).
   (iv) When $w = 0.9$, if $T^* \geq 0.1$ move to step 5.

   For individuals who remain at risk of the event at visit time $k = 1$:
5. Generate L_k conditional on $\bar{A}_{k-1}, \bar{L}_{k-1}, U, T \geq k$.
6. Generate A_k from a Bernoulli distribution conditional on $\bar{A}_{k-1}, \bar{L}_k, T \geq k$.
7. Generate event times in the period $k \leq t < k+1$ using a piecewise exponential distribution on a grid from k to $k+1$ in increments of length 0.1. First generate $V \sim \text{Uniform}(0,1)$ and

calculate $T^* = -\log(V)/\lambda(k|\bar{A}_1, \bar{L}_1, U)$. If $T^* < 0.1$ the event time is set to be $T = T^*$. If $T^* \geq 0.1$, then for $w = k + 0.1, k + 0.2, \ldots, k + 0.9$:

(i) Generate $V \sim \text{Uniform}(0, 1)$ and calculate $T^* = -\log(V)/\lambda(w|\bar{A}_k, \bar{L}_k, U)$.

(ii) If $T^* < 0.1$ the event time is set to be $T = w + T^*$.

(iii) If $T^* \geq 0.1$ move to the next value of w and return to (i).

(iv) When $w = k + 9$, if $T^* \geq 0.1$ the individual remains at risk of the event at time $k + 1$.

8. Repeat steps 5-7 for $k = 2, 3, 4$. Individuals who do not have an event time generated in the period $0 < t < 5$ are administratively censored at time 5.

References

Cole, S. and Hernán, M. (2008). Constructing inverse probability weights for marginal structural models. *American Journal of Epidemiology* **168,** 656–664.

Daniel, R., Cousens, S., De Stavola, B., Kenward, M., and Sterne, J. (2013). Methods for dealing with time-dependent confounding. *Statistics in Medicine* **32,** 1584–1618.
